# Deep Learning for AD Risk Screening and Progression Prediction

**DOI:** 10.1002/advs.78537

**Published:** 2026-11-05

**Authors:** Bin Lu, Yan‐Rong Chen, Rui‐Xian Li, Ming‐Kai Zhang, Shao‐Zhen Yan, Guan‐Qun Chen, Jing‐Hua Wang, Wen‐Hui Chai, Francisco Xavier Castellanos, Paul M. Thompson, Jie Lu, Ying Han, Chao‐Gan Yan

**Affiliations:** ^1^ State Key Laboratory of Cognitive Science and Mental Health Institute of Psychology Chinese Academy of Sciences Beijing People's Republic of China; ^2^ Department of Psychology University of the Chinese Academy of Sciences Beijing People's Republic of China; ^3^ Donders Centre for Cognitive Neuroimaging Donders Institute for Brain Cognition and Behaviour Radboud University Nijmegen the Netherlands; ^4^ Department of Psychology and Neuroscience University of North Carolina At Chapel Hill Chapel Hill North Carolina USA; ^5^ Department of Neurology National Clinical Research Center for Geriatric Diseases XuanWu Hospital of Capital Medical University Beijing People's Republic of China; ^6^ Department of Radiology and Nuclear Medicine Xuanwu Hospital Capital Medical University Beijing People's Republic of China; ^7^ Department of Neurology Beijing Chaoyang Hospital Capital Medical University Beijing People's Republic of China; ^8^ Department of Neurology of the Affiliated Hospital of Hangzhou Normal University Hangzhou People's Republic of China; ^9^ Department of Geriatrics of Changji People's Hospital Changji People's Republic of China; ^10^ Department of Child and Adolescent Psychiatry New York University Grossman School of Medicine New York New York USA; ^11^ Imaging Genetics Center Mark & Mary Stevens Institute for Neuroimaging & Informatics Keck School of Medicine University of Southern California Los Angeles California USA; ^12^ School of Biomedical Engineering Hainan University Haikou People's Republic of China; ^13^ Department of Psychological and Cognitive Sciences Tsinghua University Beijing People's Republic of China; ^14^ Research Center for Culture and Psychology Tsinghua University Beijing People's Republic of China

**Keywords:** Alzheimer's disease, deep learning, interpretability method, MRI, plasma biomarkers, progression prediction

## Abstract

The rise of Alzheimer's disease (AD) immunotherapies underscores the need for accessible, time‐efficient biomarkers for early diagnosis. MRI‐based deep learning shows promise in this regard, yet few models have undergone comprehensive clinical validation. Using the SILCODE cohort (722 Chinese participants, 1,105 scans), we test the transportability of our classifier (pretrained on Western samples) to an independent Chinese cohort without retraining. The associations between risk scores and disease progression, cognitive performance, and plasma biomarkers are examined. In addition, an interpretable framework is developed to generate individualized brain risk maps and identify subtypes. The model classifies AD with an AUC of 0.913 and 95.2% sensitivity, identifying 90.5% of high‐risk individuals 2–3 years before clinical diagnosis. Risk scores correlate significantly with cognitive and plasma indices, particularly p‐tau 217. Brain risk maps reveal AD subtypes, including a subgroup with rapid cognitive decline. These findings establish a population‐robust, clinically relevant MRI‐based model for early AD detection and risk stratification.

## Graphical abstract

A pretrained MRI‐based deep learning model generalizes from Western cohorts to an independent Chinese cohort, detects Alzheimer's disease with high sensitivity, and identifies elevated AD risk years before diagnosis. Its risk scores align with cognition and plasma biomarkers, and individualized brain risk maps uncover clinically distinct subtypes, highlighting structural MRI as a scalable route to early detection and risk stratification.

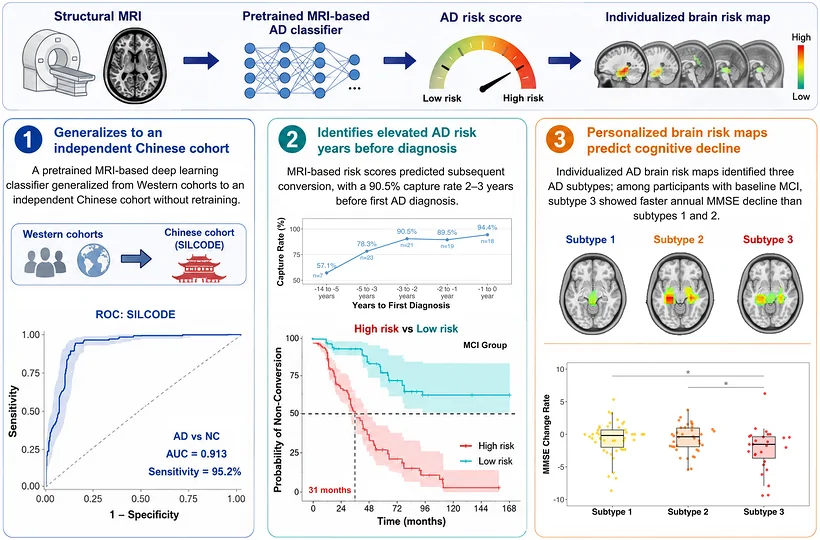

## 1 Introduction

Alzheimer's disease (AD) is a progressive neurodegenerative disease that poses a significant challenge to global health, profoundly impacting individuals, families, and healthcare systems [1]. Early detection of AD is crucial, as it allows for timely interventions that could slow disease progression and improve patient outcomes [1]. The US Food & Drug Administration recently granted traditional approval for lecanemab and donanemab for treating mild cognitive impairment (MCI) and mild AD in patients with amyloid‐beta (Aβ) proteinopathy or pathologic tau deposition [2, 3]. The advent of these novel immunotherapies highlights the need for cost‐effective and time‐efficient biomarkers for early diagnosis to enhance treatment effectiveness [4]. Traditionally, AD diagnosis has relied on observing cognitive decline, which typically occurs in later stages, often missing the critical window for effective intervention. Since biological changes often precede cognitive symptoms, integrating biomarkers from cerebrospinal fluid (CSF) and positron emission tomography (PET) into AD diagnostic guidelines has significantly enhanced early detection [5]. However, the invasive nature, high costs, and need for specially trained personnel to implement these methods restrict their widespread clinical application.

Alternatively, magnetic resonance imaging (MRI) could provide a non‐invasive and more accessible substitute. Currently, structural brain MRI is primarily used to monitor the dynamic progression of AD pathology, including staging, predicting prognosis, and patient selection and safety monitoring during drug treatment [6, 7]. However, the advent of deep learning methods has revolutionized MRI's potential by enabling the detection and use of subtle features previously unrecognizable by human experts, thus advancing the field toward earlier and more precise diagnostics and facilitating timely medical treatment [8, 9, 10]. Additionally, interpretability methods have provided initial insights into the “black box” of deep learning, potentially enhancing the confidence of physicians and patients in medical imaging models applied to clinical practice [11, 12, 13].

Despite enthusiasm for deep learning in AD diagnosis, significant limitations exist. First, robust independent validation is often lacking. Recent systematic reviews of MRI‐based AD classifiers revealed startling statistics: only 0%–5.3% of studies (e.g., 2 of 81 [8]; 0 of 16 [10]; and 6 of 114 [9]) used any independent validation dataset, let alone cross‐ethnic validation. Second, many classifiers have been trained on relatively small sample sizes, leading to models that are susceptible to overfitting. These two factors have contributed to poor generalizability, rendering many models impractical for clinical application. In response to these challenges, we employed transfer learning to construct a generalizable AD classification model based on large and diverse structural MRI datasets [14]. This classifier achieved an accuracy rate exceeding 90% across four independent AD datasets. Nevertheless, the prior study identified several areas requiring further work: first, the training and validation datasets primarily comprised Western samples, raising concerns about generalizability to other populations, such as those in China; second, the model's capability to predict disease progression needed further development, as this is essential for selecting patients with early‐stage AD for timely pharmacological treatment; third, the pathological relevance of an imaging‐based model should be validated by comparison with established biomarkers and cognitive measurements; finally, providing individualized AD‐risk brain maps may enhance the model's interpretability. Recently, plasma‐based biomarkers have shown both feasibility and high accuracy in detecting AD pathology and predicting progression of cognitive decline [15, 16]. In the 2024 revision of the NIA‐AA diagnostic criteria, plasma analytes such as phosphorylated tau 217 (p‐tau 217) immunoassay were designated as belonging to the “core‐1” category, equivalent to CSF and Amyloid PET [17]. Cross‐validating these two types of clinical feasibility biomarkers (plasma‐based and MRI‐based metrics) holds significant practical implications.

To achieve these objectives, we applied an MRI‐based classification model to a non‐Western, longitudinal, richly‐phenotyped cohort, SILCODE, which consists of a large Chinese population, assessed across the stages of the AD continuum [18]. This cohort includes extensive cognitive assessments, plasma biomarkers, and brain images. In this study, we first evaluated the transportability of the pretrained MRI‐based AD classifier to an independent Chinese cohort. Next, we extended its application to progression prediction. Subsequently, we conducted multimodal biological validation by associating model outputs with cognitive and plasma pathological indices. Finally, we introduced an interpretability method to generate individualized brain risk maps, which allowed us to identify AD subtypes and compare their cognitive decline.

## 2 Methods

### 2.1 Participants and Procedures

We included participants from the Sino Longitudinal Study on Cognitive Decline (SILCODE), a registered ongoing longitudinal clinical trial (ClinicalTrials.gov identifier: NCT02225964) focused on AD in the elderly Han population in mainland China. This study was approved by the ethics committee of Xuanwu Hospital of Capital Medical University (Approval No. 82020108013), and all participants or their guardians provided written informed consent before enrollment. The detailed SILCODE protocol has been previously described [18]. Participants were continuously recruited and followed up with flexible visit intervals, initially set at 15 months and adjusted based on cognitive evaluations administered by phone at three‐month intervals, leading to varying follow‐up times and visit intervals for participants. During each visit, participants were invited to Xuanwu Hospital for clinical evaluation, neuropsychological assessments, blood tests, and MRI scans (see Figure 1 for a schematic overview of the study design).

**FIGURE 1:**
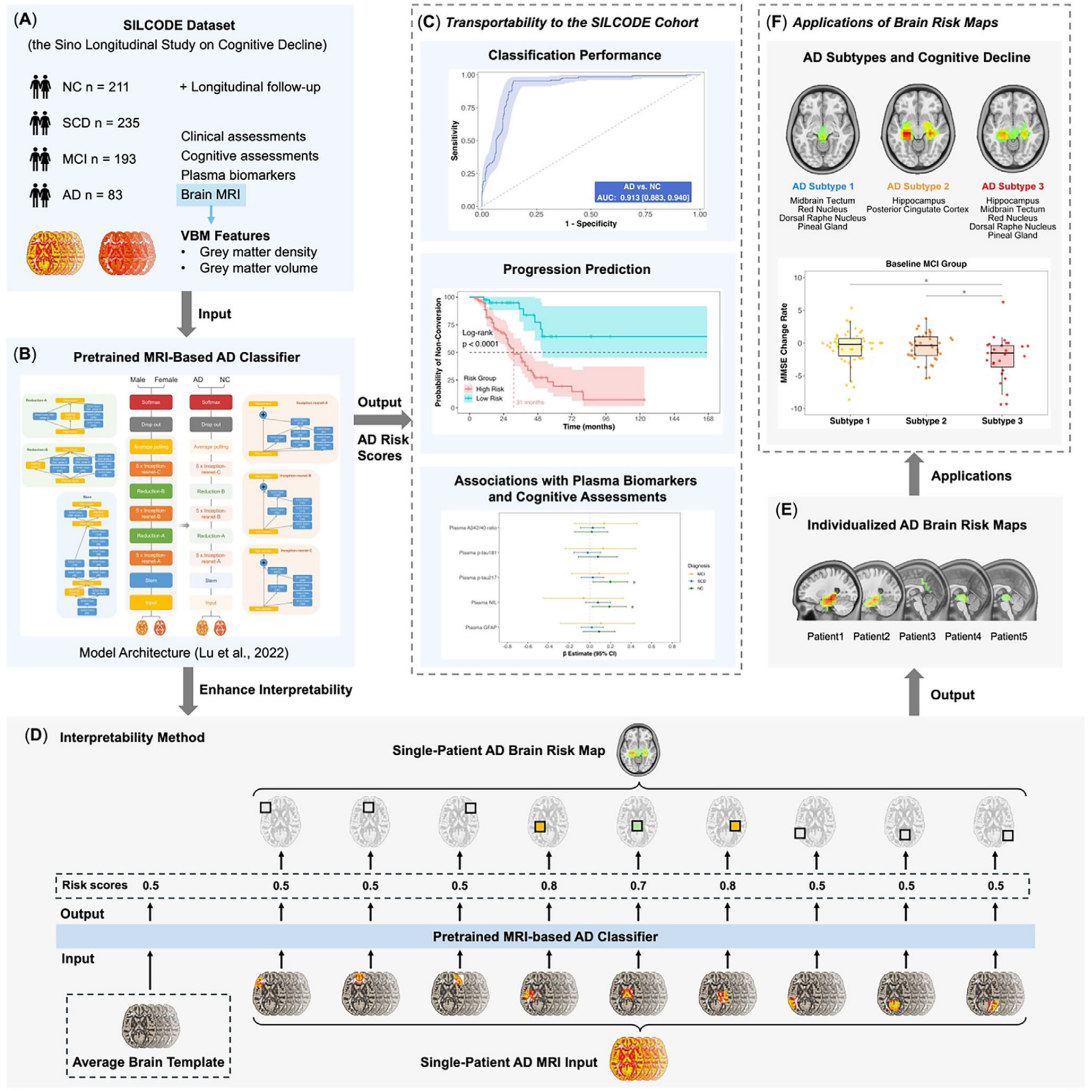
Overview of the study design. (A) SILCODE Dataset. The Sino Longitudinal Study on Cognitive Decline includes cognitively normal (NC), subjective cognitive decline (SCD), mild cognitive impairment (MCI), and Alzheimer's disease (AD) participants with longitudinal follow‐up. Clinical assessments, cognitive measurements, plasma biomarkers, and structural MRI were collected. Voxel‐based morphometry (VBM) derived grey matter density and volume maps were used as imaging features. (B) Pretrained MRI‐Based AD Classifier. A previously developed deep learning classifier [14] was adapted to the SILCODE cohort without any retraining or fine‐tuning. (C) Transportability to an independent Chinese cohort. The model pretrained on North American MRI cohorts was externally evaluated on the SILCODE dataset through three complementary analyses: (Top) AD–NC classification performance, (Middle) prediction of clinical progression in baseline MCI, and (Bottom) associations between model‐derived AD risk scores, plasma biomarkers, and cognitive measures. (D) Interpretability Method. Each brain region of an individual's MRI was sequentially substituted into a neutral average template whose predicted AD score was calibrated to 0.5. This procedure yielded region‐specific AD risk scores, which were then combined to generate the individualized AD brain risk map. (E) Individualized AD Brain Risk Maps. For each participant, a personalized brain risk map highlights spatial patterns contributing to higher AD likelihood. (F) Example Applications of Brain Risk Maps. AD subtype analysis reveals distinct MCI subgroups with different cognitive decline rates. AD, Alzheimer's disease; VBM, voxel‐based morphometry.

The current study initially included 738 participants, all over age 50 years at the time of their first MRI scan, who had completed at least one MRI session. After quality control, 16 participants were excluded due to invalid MRI scans, leaving a final sample of 722 participants (60.8% female; mean age = 66.1 ± 7.7 years at baseline). Diagnostic distribution at baseline was: 211 normal cognition (NC), 235 subjective cognitive decline (SCD), 193 MCI, and 83 AD. Participants and their visit sessions were further filtered based on the requirements of specific analyses.

### 2.2 Diagnostic Criteria

During each visit, participants were classified into AD, MCI, SCD, and NC groups, with re‐evaluations in subsequent visits to detect changes [18]. AD diagnosis followed NIA‐AA guidelines [19], supplemented by a global Clinical Dementia Rating [20] (CDR) score of at least 1. MCI was diagnosed using the Jak/Bondi method [21] with neuropsychological tests (see below) and thresholds adjusted for the Chinese population [18]. SCD classification followed Jessen et al.’s criteria [22] for SCD plus. NC was assigned to those without subjective or informant‐reported memory decline or evidence of cognitive impairment. In overlapping cases, the more severe condition was designated. All clinical diagnoses were made by qualified physicians.

### 2.3 Neuropsychological Tests

A battery of neuropsychological tests was used to measure cognitive levels of participants, focusing on both global domain and specific subdomains including memory, language, and executive functions. Global cognitive function was assessed using the Mini‐Mental State Examination [23] (MMSE), Memory and Executive Screening [24] (MES), and the Montreal Cognitive Assessment Basic [25] (MOCA‐B). For specific subdomains, memory tests included the Auditory Verbal Learning Test—Huashan version [26]—Long delayed memory (AVLT‐L) and Recognition (AVLT‐R); the language tests comprised the Animal Fluency Test [27] (AFT), and 30‐item Boston Naming Test [28] (BNT); and executive functions were evaluated using the Shape Trail Test [29] Parts A (STT‐A) and B (STT‐B). All cognitive assessments were administered by trained neuropsychologists.

### 2.4 Biological Tests

Plasma levels of Aβ42, Aβ40, NfL, GFAP, p‐tau 217, and p‐tau 181 were measured by immunoassay using a Simoa HD‐X analyzer (Quanterix, Billerica, MA, USA), as per the manufacturer's protocols. Specifically, plasma samples were thawed at room temperature and then centrifuged for 5 min at 4°C and 10 000 × g. The resultant supernatant was transferred to 96‐well plates and diluted four‐fold with sample diluent, followed by a two‐step digital immunoassay. The concentrations of Aβ42, Aβ40, NfL, and GFAP were measured with the Simoa Neurology 4‐Plex E Advantage Kit (Quanterix, Billerica, MA, USA; Cat# 103670). The ALZpath Simoa pTau‐217 v2 Assay Kit (Quanterix, Cat# 104371) was used to measure p‐tau 217, and the Simoa pTau‐181 Advantage V2 Kit (Quanterix, Cat# 103714) was used to measure p‐tau 181. Assays were performed using kits from the same lot, with all samples run in duplicate. Seven or eight calibrators (2x) and two quality controls (2x) were run on each plate for each analyte. The lower limits of detection were 0.136 pg/mL for Aβ42, 0.384 pg/mL for Aβ40, 0.090 pg/mL for NfL, 0.441 pg/mL for GFAP, 0.019 pg/mL for p‐tau 217, and 0.028 pg/mL for p‐tau 181. The lower limits of quantification were 0.378 pg/mL for Aβ42, 1.02 pg/mL for Aβ40, 0.400 pg/mL for NfL, 2.89 pg/mL for GFAP, 0.061 pg/mL for p‐tau 217, and 0.338 pg/mL for p‐tau 181. The intra‐assay coefficients of variation for controls ranged from 2% to 13% for Aβ42, 1% to 5% for Aβ40, 2% to 12% for NfL, 1% to 8% for GFAP, 2% to 11% for p‐tau 217, and 1% to 10% for p‐tau 181.

### 2.5 MRI Acquisition and Preprocessing

T1‐weighted structural brain MRI data were collected using a single 3.0 T GE SIGNA PET/MR scanner (GE Healthcare, Milwaukee, WI, USA) at Xuanwu Hospital of Capital Medical University. Scanning parameters were as follows: SPGR sequence with inversion preparation, repetition time (TR) = 6.9 ms, echo time (TE) = 2.98 ms, inversion time (TI) = 450 ms, slice number = 192, slice thickness = 1 mm, gap = 0, flip angle = 12°, field of view (FOV) = 256 × 256 mm^2^, matrix = 256 × 256, voxel size = 1 × 1 × 1 mm^3^.

Manual quality control was conducted to exclude low‐quality images with notable artifacts or incomplete brain coverage. Images were then preprocessed using the sMRI preprocessing pipeline from the Data Processing Assistant for Resting‐State fMRI (DPARSF) Toolbox. Specifically, we processed individual T1‐weighted images using the voxel‐based morphometry (VBM) module in DPARSFA, which is built upon SPM [30]. Each image was segmented into grey matter, white matter, and cerebrospinal fluid. The segmented tissue maps were then spatially normalized from native space to the MNI‐152 template [31] using the Diffeomorphic Anatomical Registration Through Exponentiated Lie Algebra (DARTEL) algorithm [32]. The derived grey matter density (GMD) and grey matter volume (GMV) maps were subsequently extracted and used as input features for the AD deep learning model.

### 2.6 AD Deep Learning Classifier Training and Risk Score Generation

The AD deep learning model employed in this study is the transfer learning–based classifier developed in our previous work [14]. The network adopts an Inception‐ResNet‐V2 architecture optimized for 3D MRI data, comprising a stem module, three convolutional modules, and two reduction modules. The model was first pretrained on T1‐weighted MRI samples acquired from more than 200 sites/scanners to enhance its ability to extract and leverage brain structural features. It was then fine‐tuned for AD classification using 6856 MRI scans from approximately 1078 participants from the Alzheimer's Disease Neuroimaging Initiative (ADNI). Importantly, to improve robustness and minimize potential site‐specific biases, a leave‐sites‐out cross‐validation strategy—rather than random cross‐validation—was used to train and validate the model. The resulting AD classifier was further evaluated in three external datasets (2436 scans from 722 participants). Across all four datasets, the model consistently achieved over 90% accuracy and an AUC above 0.96, demonstrating strong classification performance and generalizability.

However, the original model was trained and tested on the United States, Canada, and Australia populations, without cross‐ethnic validation in more divergent populations. Therefore, in the present study, we directly applied this pretrained AD classifier to a Chinese AD dataset (SILCODE) to evaluate its transportability to an independent Chinese cohort and clinical applications. Notably, the model was applied without any fine‐tuning or parameter adjustment on the SILCODE data, thereby preventing information leakage and ensuring an unbiased assessment of generalization. For the purpose of facilitating downstream statistical analysis and clinical interpretation, we refer to the classifier's output probability as the AD risk score.

Depending on the type of downstream analysis, two forms of risk scores were used in this study: sigmoid‐based scores and linear scores. Sigmoid‐based risk scores were obtained using sigmoid activation at the output layer in the deep learning model, as per the previous AD classifier paper [14]. The sigmoid‐based risk score ranges from 0 to 1, making it easy to interpret and suitable for defining decision thresholds. It is commonly used for classification tasks. On the other hand, we also calculated linear risk scores using linear activation at the output layer in the deep learning model. These scores were included because their distribution is closer to a normal distribution, making them more suitable for statistical testing such as linear mixed‐effects modeling.

### 2.7 Statistical Analysis

We first evaluated the performance of the MRI‐based AD classifier on the Chinese SILCODE cohort using receiver operating characteristic (ROC) curve analysis. The area under the curve (AUC) was calculated, with 95% confidence intervals (CIs) estimated using 5000 bootstrap samples. The optimal cutoff score was determined using the Youden index [33]. At this threshold, we calculated accuracy, sensitivity, specificity, positive predictive value (PPV), and negative predictive value (NPV), with corresponding 95% CIs to assess classifier performance. To rule out potential subject‐weighting bias from participants with multiple visits, we additionally performed a supplementary subject‐level validation using the last available scan for each participant (Method S1). As an exploratory sensitivity analysis, we conducted an age‐matched evaluation, in which the SILCODE AD and NC groups were separately matched to the corresponding AD and NC groups in the original training cohort [14], to contextualize classification performance (Methods S2). We additionally evaluated classification performance using a cutoff of 0.50 alongside the Youden‐index‐derived cutoff of 0.38. We further assessed calibration to evaluate the agreement between predicted and observed probabilities, using the calibration intercept, calibration slope, and Brier score, with calibration plots used to visualize calibration performance.

Next, we applied a linear mixed‐effects model to examine the association between session‐level classifier linear scores (as the dependent variable) and clinical diagnoses (NC, SCD, MCI, AD; as the predictor), controlling for sex, age, and education. Participants were included as a random intercept to account for within‐subject correlation across repeated measures. The Satterthwaite approximation was used to adjust degrees of freedom for fixed effects, accounting for the uncertainty introduced by random factors. Post‐hoc pairwise comparisons were conducted using estimated marginal means with Tukey correction to examine differences between diagnostic groups. Complementary subject‐level analyses using the last scan were also conducted to verify the consistency of these associations.

Survival analyses were conducted to examine the probability of progression to AD in NC, SCD, and MCI participants. Participants were divided into high‐risk and low‐risk groups based on AD classifier risk scores at baseline, with the cutoff (i.e., 0.38) recalibrated in the SILCODE cohort through ROC analysis using the Youden index. Kaplan‐Meier survival curves were generated to illustrate the probability of participants maintaining non‐AD status over the follow‐up period, with the log‐rank test comparing survival distributions between groups. To further quantify the predictive value, we employed Cox proportional hazards regression using continuous standardized baseline linear risk scores to assess the hazard of conversion. Both unadjusted and adjusted models were examined in the full sample and by diagnostic subgroup, with adjusted models controlling for age, sex, and education. In the full sample, baseline diagnosis was additionally included. Because no NC participants converted to AD, NC and SCD were combined as the reference category to avoid unstable estimates. This variable violated the proportional hazards assumption and was therefore included as a stratification variable. The proportional hazards assumption was assessed using Schoenfeld residuals. Given only three conversion events in the SCD subgroup, only the unadjusted model was reported.

To assess how early the classifier could predict conversion, we analyzed all pre‐diagnosis visit sessions from AD converters. Specifically, we calculated the capture rate at different time intervals, defined as the proportion of high‐risk sessions (score ≥ 0.38) pooled from all converters within each interval. Mean risk scores were also computed to observe trends in AD risk over time. Due to the limited data, particularly the decrease in data size as the time before diagnosis increased, we categorized time intervals to simplify analyses. Intervals were set as within 1 year, 1–2 years, 2–3 years, 3–5 years, and 5–14 years before the first AD diagnosis (Figure S1). To capture the gradual temporal evolution of AD risk relative to disease proximity, a complementary linear mixed‐effects model was fitted with the linear risk score as the dependent variable and years‐to‐diagnosis as a continuous predictor (expressed as negative values, with larger values representing a time closer to diagnosis). Participants were included as a random intercept to account for repeated measures. The model controlled for sex and education (age was excluded from the covariates due to its high collinearity with years‐to‐diagnosis).

To examine the associations of MRI‐based risk scores with cognitive tests and plasma biomarkers, separate models were fitted for each measure. Cognitive and plasma outliers exceeding six times the interquartile range (6IQR) [34] were excluded to minimize their influence. Global associations were first assessed across the entire sample to identify general patterns, followed by diagnosis‐specific analyses within pre‐AD phases (NC, SCD, MCI) to explore phase‐specific associations critical for early detection and intervention. Linear mixed‐effects models were generally used, with the linear risk score as the independent variable, sex, age, and education as covariates, and participant included as a random intercept to account for within‐subject correlation across repeated measures. All continuous variables, including the dependent variables to facilitate comparison across different outcomes, were standardized, and sex was sum‐coded. However, for plasma biomarker models within the MCI group, general linear models without the random effect were applied due to the limited number of repeated measures per participant (e.g., 34 participants had only 37 plasma p‐tau 217 measures), which caused convergence issues in mixed‐effects models. Effect sizes were measured using Cohen's *f^2^*, which reflects the extent to which the inclusion of a specific predictor variable improves the model's explanatory power. The Satterthwaite approximation was used to adjust degrees of freedom for fixed effects in mixed‐effects models. Post‐hoc pairwise comparisons were performed using the estimated marginal means with Tukey correction in all models. To examine whether associations between the risk score and outcomes differed by diagnostic stage and demographic characteristics, we additionally conducted interaction analyses examining sex, age, education, and diagnosis stage as potential moderators, with details provided in Method S3 and Tables S2 and S3.

Differences across brain subtypes (see the next section for details on identification) in variables of interest, such as MMSE decline rate, baseline MMSE level, baseline linear risk score, and demographic variables, were assessed using ANOVA, followed by post‐hoc pairwise comparisons of estimated marginal means with LSD correction.

All analyses and visualizations were conducted using R 4.4.0 [35], along with the following packages: pROC [36] (for ROC analysis), lme4 [37] and lmerTest [38] (for linear mixed models), survival [39] and survminer [40] (for survival analysis), and emmeans [41] (for post‐hoc comparisons).

### 2.8 Identifying Brain Regions at High Risk for AD

We developed an interpretability method that enables individualized identification of brain regions at risk for AD (Figure 1D). Overall, we constructed a neutral reference brain template and applied regional perturbation to estimate each region's contribution to AD risk for an individual patient. The detailed procedure is described below:

First, we constructed a reference brain template whose predicted AD probability was approximately 0.5, representing an intermediate state between typical AD (risk score = 1) and NC (risk score = 0). This template served as a neutral reference in our interpretability approach, allowing us to quantify the contribution of each brain region to AD risk through controlled perturbation experiments. Constructing a neutral reference template—rather than a typical NC or AD template—ensured maximal sensitivity to AD‐related variations. Because the model's output layer uses a sigmoid activation function, its gradient is highest near 0.5, and thus its sensitivity to small input perturbations is greatest. To obtain this template, we performed 10 000 random samplings from AD and NC participants in the ADNI dataset. In each sampling, 100 participants were randomly selected, and their GMD and GMV maps were averaged to form candidate templates. The pre‐trained MRI‐based AD classifier was then used to predict the AD risk score for each candidate template, and the template whose predicted score was closest to 0.5 (0.499928 in the current study) was chosen as the final reference.

Next, we performed a regional perturbation analysis to estimate the contribution of each brain region to the AD risk of an individual participant. Starting from the reference template, we sequentially replaced one region at a time with the participant's own GMD and GMV values. Each region was defined as a cube with an edge length of 18 mm, moved across the brain with a step size of 12 mm. For every replacement, the modified image was input into the classifier to obtain a new AD risk score. Increases in the predicted AD risk scores (e.g., from 0.5 to 0.88 after inserting an AD patient's left hippocampal region) indicated that the corresponding region strongly promoted AD probability for that participant, whereas decreases toward 0 suggested relatively preserved structure. Repeating this procedure across all regions and reprojecting the resulting scores back onto the brain yielded an individualized AD brain risk map for each subject.

### 2.9 Brain Risk Map‐Based Subtypes and Their Distinct Cognitive Decline Rates

We used the individualized brain risk maps to identify distinct AD subtypes and to assess the differential rates of cognitive decline across these subtypes. Specifically, we employed k‐means cluster analysis using the brain risk maps of AD patients in the SILCODE dataset as features. We selected k‐means because it provides a simple and transparent clustering procedure that can be directly applied to the individualized MRI‐based risk maps. K‐means clustering was performed using MATLAB's built‐in implementation with the default settings, including k‐means++ initialization, a maximum of 100 iterations, and a convergence tolerance of 1 × 10^−^^4^. Euclidean distance was used as the similarity metric, and the algorithm iteratively updated cluster centroids until changes in centroid positions fell below the convergence threshold. We determined the optimal number of clusters for k‐means clustering by employing the elbow method and silhouette width analysis. The elbow method utilizes the sum of squared errors (SSE) to identify the point at which the increase in the number of clusters does not result in significantly better modeling of the data. Silhouette width analysis evaluates clustering quality by quantifying how well each observation matches its assigned cluster relative to neighboring clusters, with higher values indicating better within‐cluster cohesion and between‐cluster separation. Cluster stability across repeated initialization was assessed by rerunning k‐means with different random initializations and quantifying the consistency of the resulting cluster assignments. The centroid of each cluster derived from the k‐means analysis reflects the characteristic brain risk pattern defining that AD subtype. Each patient was assigned to an AD subtype by computing the spatial correlation between the patient's brain risk map (derived from the baseline MRI scan) and the prototypical pattern of each AD subtype (i.e., the cluster centroid).

Subsequently, we examined differences in cognitive decline rates across the brain risk map‐based subtypes. Given that MCI represents the stage with the most pronounced cognitive decline, this analysis focused primarily on individuals with baseline MCI diagnoses (*n* = 111). The rate of cognitive decline was defined as the annual change in MMSE score, calculated as the difference between the baseline and most recent MMSE score divided by the number of follow‐up years. A general linear model was used to compare the annual change in MMSE score across subtypes, adjusting for sex, age, and education, but not for baseline MMSE score, cognitive performance, or duration of follow‐up.

## 3 Results

### 3.1 Study Participants

The current study included 722 participants from the SILCODE dataset (60.8% female; 66.1 ± 7.7 years of age at baseline), categorized based on clinical assessments at baseline into 211 NC, 235 SCD, 193 MCI, and 83 AD participants. They completed 1980 visits and provided 1105 valid MRI scans. Table 1 presents descriptive data for these MRI sessions (60.8% female; 66.6 ± 7.6 years; 348 NC, 363 SCD, 269 MCI, 125 AD), which serve as the basis for the following validations of classificatory ability and pathological value of the MRI‐based deep learning model.

**TABLE 1:** Descriptive Data for Visit Sessions with Valid MRI Scans.

	ALL (*n* = 1105)	NC (*n* = 348)	SCD (*n* = 363)	MCI (*n* = 269)	AD (*n* = 125)
**Female, *n* (%)**	672 (60.81%)	197 (56.61%)	240 (66.12%)	154 (57.25%)	81 (64.80%)
**Age (years)**	66.60 ± 7.65	64.91 ± 6.52	66.18 ± 6.29	67.51 ± 8.70	70.55 ± 9.84
**Education (years)**	11.30 ± 4.24	11.92 ± 3.75	12.29 ± 3.53	9.97 ± 4.65	9.58 ± 5.17
**Risk score**	0.40 ± 0.33	0.21 ± 0.24	0.36 ± 0.30	0.55 ± 0.32	0.72 ± 0.23
**Linear risk score**	−0.76 ± 2.52	−2.18 ± 2.02	−1.03 ± 2.21	0.30 ± 2.38	1.73 ± 2.02
**MMSE**	26.14 ± 4.75 (*n* = 1053)	28.63 ± 1.54 (*n* = 330)	28.32 ± 1.67 (*n* = 337)	24.42 ± 3.80 (*n* = 266)	17.00 ± 5.57 (*n* = 120)
**MES**	88.35 ± 10.91 (*n* = 492)	91.29 ± 6.93 (*n* = 188)	90.78 ± 6.54 (*n* = 241)	75.65 ± 10.91 (*n* = 49)	51.29 ± 15.90 (*n* = 14)
**MOCA‐B**	24.56 ± 4.21 (*n* = 539)	26.12 ± 2.51 (*n* = 198)	25.61 ± 2.53 (*n* = 253)	20.06 ± 3.43 (*n* = 67)	11.67 ± 3.73 (*n* = 21)
**AVLT‐L**	6.60 ± 2.82 (*n* = 540)	7.58 ± 2.12 (*n* = 198)	7.29 ± 2.12 (*n* = 254)	3.00 ± 2.08 (*n* = 69)	0.26 ± 0.81 (*n* = 19)
**AVLT‐R**	21.64 ± 2.72 (*n* = 538)	22.49 ± 1.68 (*n* = 199)	22.31 ± 1.70 (*n* = 253)	18.51 ± 2.95 (*n* = 69)	14.41 ± 4.05 (*n* = 17)
**AFT**	18.13 ± 4.91 (*n* = 509)	19.08 ± 4.19 (*n* = 191)	18.84 ± 4.62 (*n* = 245)	14.91 ± 3.97 (*n* = 55)	8.17 ± 3.52 (*n* = 18)
**BNT**	24.47 ± 3.72 (*n* = 509)	25.59 ± 2.96 (*n* = 191)	24.86 ± 3.07 (*n* = 245)	21.40 ± 3.67 (*n* = 55)	16.83 ± 5.28 (*n* = 18)
**STT‐A**	65.43 ± 28.37 (*n* = 506)	58.72 ± 18.15 (*n* = 191)	60.77 ± 21.81 (*n* = 245)	91.99 ± 34.28 (*n* = 54)	127.44 ± 58.91 (*n* = 16)
**STT‐B**	150.41 ± 55.51 (*n* = 501)	137.95 ± 38.66 (*n* = 191)	139.47 ± 43.33 (*n* = 244)	220.77 ± 75.18 (*n* = 53)	251.85 ± 65.56 (*n* = 13)
**Plasma Aβ42/40 ratio**	0.06 ± 0.01 (*n* = 377)	0.06 ± 0.01 (*n* = 132)	0.06 ± 0.01 (*n* = 199)	0.05 ± 0.01 (*n* = 34)	0.04 ± 0.01 (*n* = 12)
**Plasma p‐tau 181 (pg/ml)**	2.13 ± 1.03 (*n* = 361)	1.90 ± 0.81 (*n* = 124)	1.99 ± 0.83 (*n* = 194)	3.20 ± 1.50 (*n* = 32)	3.96 ± 1.27 (*n* = 11)
**Plasma p‐tau 217 (pg/ml)**	0.34 ± 0.24 (*n* = 341)	0.27 ± 0.11 (*n* = 112)	0.32 ± 0.19 (*n* = 184)	0.52 ± 0.37 (*n* = 37)	1.06 ± 0.26 (*n* = 8)
**Plasma *Nf*L (pg/ml)**	19.57 ± 13.21 (*n* = 376)	17.90 ± 12.02 (*n* = 131)	18.95 ± 12.91 (*n* = 202)	26.85 ± 17.28 (*n* = 31)	29.58 ± 9.36 (*n* = 12)
**Plasma GFAP (pg/ml)**	104.38 ± 62.35 (*n* = 379)	85.75 ± 40.66 (*n* = 132)	99.99 ± 50.07 (*n* = 203)	148.46 ± 86.58 (*n* = 32)	266.19 ± 89.47 (*n* = 12)

Abbreviations: NC, normal cognition; SCD, subjective cognitive decline; MCI, mild cognitive impairment; AD, Alzheimer's disease; Risk score, MRI‐based classifier's output score; Linear risk score, MRI‐based classifier's linear activation output score; MMSE, Mini‐Mental State Examination; MES, Memory and Executive Screening Score; MOCA‐B, Montreal Cognitive Assessment‐Basic; AVLT‐L, Auditory Verbal Learning Test‐Long delayed memory; AVLT‐R, Auditory Verbal Learning Test‐Recognition; AFT, Animal Fluency Test; BNT, Boston Naming Test; STT‐A, Shape Trail Test ‐ Part A; STT‐B, Shape Trail Test ‐ Part B; NfL, neurofilament light chain; GFAP, glial fibrillary acidic protein. NOTE: All measurements are reported as mean ± s.d., unless otherwise specified. If a measurement does not include all samples, the actual sample size is listed in parentheses.

### 3.2 AD Classification Performance in a Chinese Sample

When directly applied to the SILCODE cohort without any re‐training or fine‐tuning, the MRI‐based AD classifier demonstrated robust performance in distinguishing AD from NC. As Figure 2A shows, the area under the curve (AUC) was 0.913 (95% CI = [0.883, 0.940]), indicating solid discriminative capability. The calibration results are shown in Figure S8. An optimal cutoff score of 0.38 was identified using the Youden index. At this threshold, the classifier achieved an accuracy of 88.2% (95% CI = [84.9%, 90.9%]), a sensitivity of 95.2% (95% CI = [89.8%, 98.2%]), a specificity of 85.6% (95% CI = [81.5%, 89.1%]), a positive predictive value (PPV) of 70.4% (95% CI = [62.9%, 77.2%]), and a negative predictive value (NPV) of 98.0% (95% CI = [95.8%, 99.3%]). Classification performance using a cutoff score of 0.5 is shown in Table S1. To rule out potential subject‐weighting bias, we also conducted a subject‐level analysis, which yielded a nearly identical AUC of 0.909 (95% CI = [0.876, 0.940]) (Figure S2A). In an exploratory analysis using a SILCODE subset age‐matched to the training cohorts, the classifier yielded an AUC of 0.962 (95% CI = [0.917, 0.992]) (Figure S3), although the sample size was limited.

**FIGURE 2:**
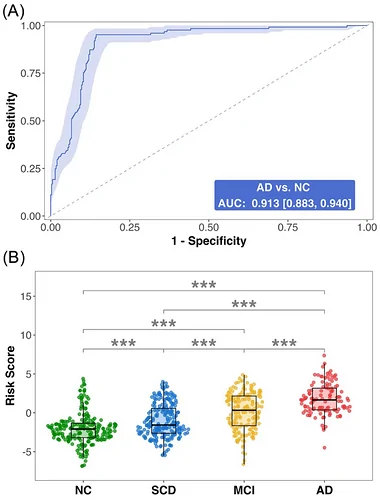
Classification performance of the MRI‐based AD classifier on the SILCODE Cohort. (A) The receiver operating characteristic (ROC) curve shows the classifier's performance in distinguishing Alzheimer's disease (AD) from normal cognition (NC), with an area under the curve (AUC) of 0.913 (95% confidence interval [CI]: 0.883, 0.940). The shaded area represents the 95% CI. (B) Box plots show the median, interquartile range, and whiskers extending 1.5 times the interquartile range, with individual data points displayed. ^***^ *p* < 0.001.

### 3.3 Classifier Scores Correspond to Clinical Diagnoses

Adjusting for sex, age, and education, and accounting for non‐independent samples by including participant as a random effect, we found that linear risk scores^1^ varied significantly by clinical diagnoses (*F*(3, 1058.04) = 73.96, *p* < 2.20 × 10^−16^). Post‐hoc Tukey‐corrected comparisons revealed that the AD group had significantly higher scores than NC (mean difference = 3.36, *SE* = 0.24, *t*(1018.86) = 14.11, *p* = 7.85 × 10^−13^), SCD (mean difference = 2.30, *SE* = 0.23, *t*(1045.43) = 9.84, *p* < 2.20 × 10^−16^), and MCI groups (mean difference = 1.23, *SE* = 0.22, *t*(1051.45) = 5.61, *p* = 1.57 × 10^−7^). MCI also scored higher than NC (mean difference = 2.13, *SE* = 0.19, *t*(947.90) = 11.10, *p* = 3.35 × 10^−13^) and SCD (mean difference = 1.07, *SE* = 0.19, *t*(1036.81) = 5.78, *p* = 6.05 × 10^−8^). In addition, SCD scored higher than NC (mean difference = 1.06, *SE* = 0.17, *t*(1038.16) = 6.78, *p* = 6.05 × 10^−8^). As shown in Figure 2B, the relative ordering of scores across diagnostic groups aligned with expected severity along the AD continuum, despite the classifier not being trained or fine‐tuned on SCD or MCI data. These findings were consistently replicated in a subject‐level analysis (Figure S2B).

### 3.4 Performance In Predicting Disease Progression

A total of 474 participants, comprising 124 with MCI, 195 with SCD, and 155 with NC as diagnosed at their baseline visits, who completed at least two visit sessions, were included in the survival analysis. Participants were further classified as high‐risk (risk score ≥ 0.38) or low‐risk (risk score < 0.38) based on their baseline sigmoid risk scores. In the full sample, 60 participants converted to AD during the 167‐month follow‐up period, including 57 conversions from the baseline MCI group and 3 from the baseline SCD group. No baseline NC participant converted to AD during the tracking period. Kaplan‐Meier survival plots illustrate the detailed progression of AD conversion in the full sample (Figure 3A), baseline MCI group (Figure 3B), and baseline SCD group (Figure 3C). Notably, as shown in the table at the bottom of Figure 3A, our model identified 52 of 60 AD converters as high risk at baseline, achieving an overall capture rate of 86.7%. In the full sample, 52 of the 177 participants classified as high‐risk and 8 of the 297 participants classified as low‐risk converted to AD during the follow‐up period. The log‐rank test indicated a significant difference between the survival curves (*p* < 0.0001), accounted for by higher probability of conversion for participants in the high‐risk group.

**FIGURE 3:**
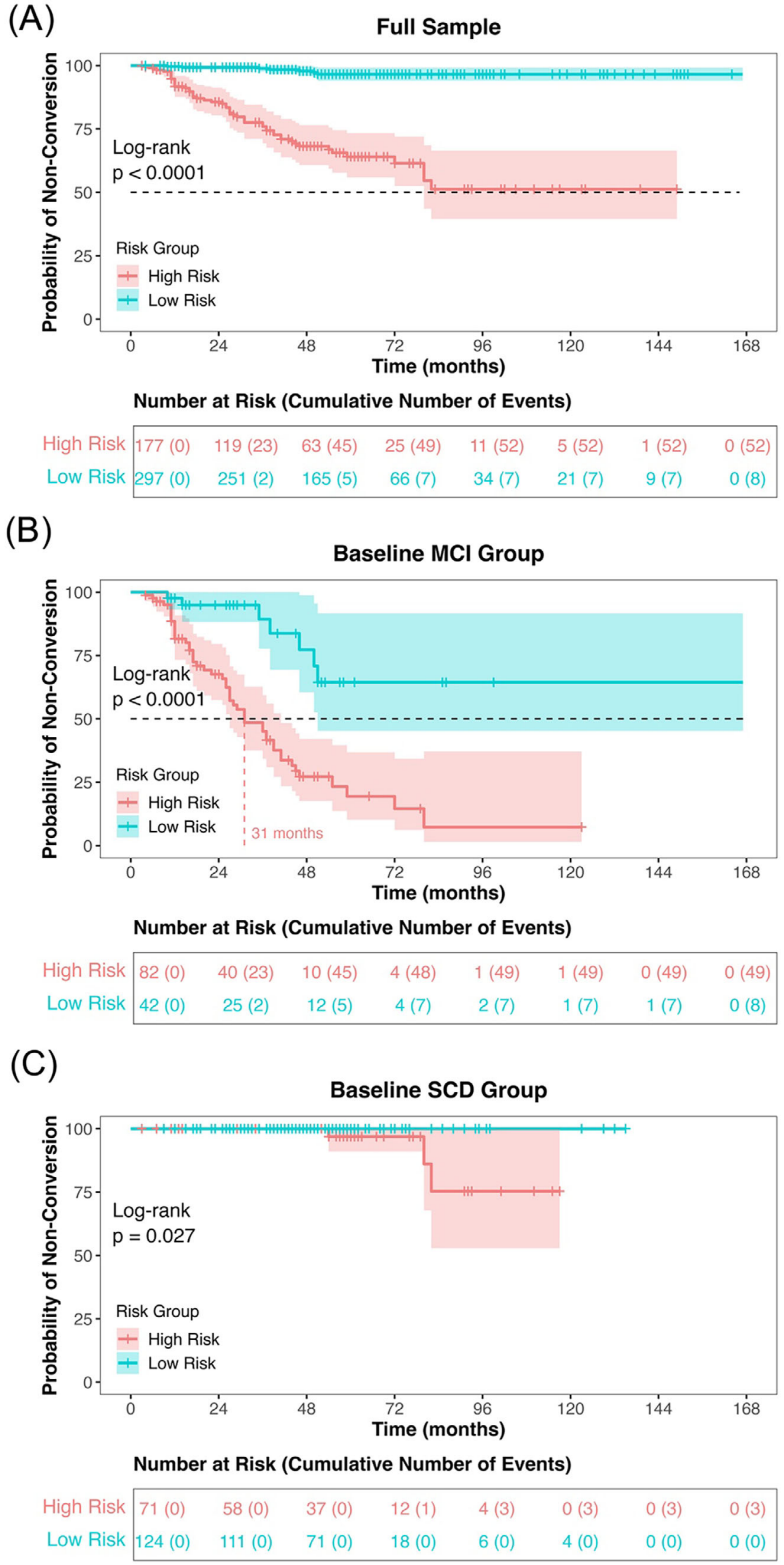
Survival analysis of clinical conversion in the full cohort and baseline diagnostic subgroups (MCI and SCD). Kaplan‐Meier survival (conversion) curves showing the probability of non‐conversion in high‐risk (red) and low‐risk (blue) groups over 167 months (the longest follow‐up period). High risk was defined by baseline AD sigmoid risk scores (≥0.38). Curves are shown for the full sample (A), MCI (B), and SCD (C) at baseline. The baseline NC sample is not shown, as no baseline NC participant progressed to AD during the tracking period. Shaded areas represent 95% confidence intervals, with vertical ticks indicating censored participants (lost to follow‐up). Vertical dotted lines mark median conversion times, and the horizontal black dashed line indicates a 50% probability of non‐conversion. Log‐rank tests compared survival (conversion) distributions between the two groups, with *p* < 0.05 indicating significance. Tables below the plots show the number of participants at risk (i.e., still followed and non‐progressed) at each time point, with cumulative conversion events in parentheses. To avoid confusion, the survival curves for the low‐risk group (A and B) omit the abrupt drop to zero at the tail caused by the last single individual's conversion to AD. MCI, mild cognitive impairment; SCD, subjective cognitive decline.

Specifically, among participants with MCI at baseline (Figure 3B), 49 of the 82 high‐risk participants converted to AD, compared to 8 of the 42 low‐risk participants, showing a significantly higher probability of conversion to AD in the high‐risk group (log‐rank test, *p* < 0.0001). The median survival time (i.e., time‐to‐conversion) of the high‐risk MCI group (31 months) was significantly shorter than that of the low‐risk MCI group (> 167 months). In participants with SCD at baseline, only 3 conversions occurred, and all were in the high‐risk category (Figure 3C). The log‐rank test indicated a significantly higher risk of conversion for high‐risk SCD participants (*p* = 0.027, Figure 3C).

To further quantify the progression predictive value of the risk score, we performed Cox proportional hazards regression using standardized continuous linear risk scores. In the full cohort, the risk score was significantly associated with the hazard of conversion in the unadjusted model (HR = 2.97, 95% CI [2.27–3.89], *p* = 2.27 × 10^−^^1^ ^5^), indicating that each 1 SD increase in baseline risk score was associated with approximately a 197% higher hazard of conversion. This association was attenuated but remained significant after adjusting for age, sex, education, and stratified baseline diagnosis (HR = 1.58, 95% CI [1.17–2.13], *p* = 003), corresponding to an approximately 58% higher hazard per 1‐SD increase in risk score. In the MCI subgroup, each 1 SD increase in risk score was associated with a 68% higher hazard of conversion in the unadjusted model (HR = 1.68, 95% CI [1.26–2.23], *p* <.001), and a 54% higher hazard after adjustment (HR = 1.54, 95% CI [1.13–2.09], *p* = 006). In the SCD subgroup, the risk score showed a similar direction of association with conversion hazard (HR = 1.91, 95% CI [0.67–5.45], *p* = .227), corresponding to an approximately 91% higher hazard per 1‐SD increase in risk score, although the association did not reach statistical significance, likely due to limited statistical power. Given the small number of conversion events (*n* = 3), only the unadjusted model was reported because stable multivariable estimation was not feasible. The proportional hazards assumption was satisfied for all final models.

In summary, both survival and Cox regression analyses demonstrated that higher baseline risk scores were associated with a higher risk of subsequent conversion to AD, with the association remaining significant after adjustment for demographic and diagnostic factors in the Cox models.

### 3.5 Classifier Predicted AD Progression Years Before Diagnosis

Using all visit sessions (*n* = 88) from the 60 participants who progressed from NC, SCD, or MCI, we estimated *how early* the classifier was able to predict AD conversion. Specifically, we calculated the ratio of sessions flagged as high risk by the classifier (risk score ≥ 0.38) at various time intervals before the first AD diagnosis. Results (Figure 4A) show that the classifier achieved a capture rate of about 90.5% within 2–3 years, 78.3% within 3–5 years, and 57.1% within 5–14 years before the first AD diagnosis among AD converters. Mean risk scores increased monotonically across follow‐up periods, consistent with the probability of conversion (Figure 4B). To assess AD risk score changes relative to disease proximity, a linear mixed‐effects model was fitted with risk scores as the dependent variable and years‐to‐diagnosis as the continuous predictor. The results revealed a positive relationship (*β* = 0.41, *p*<0.001), which means that risk scores increased as participants approached clinical diagnosis.

**FIGURE 4:**
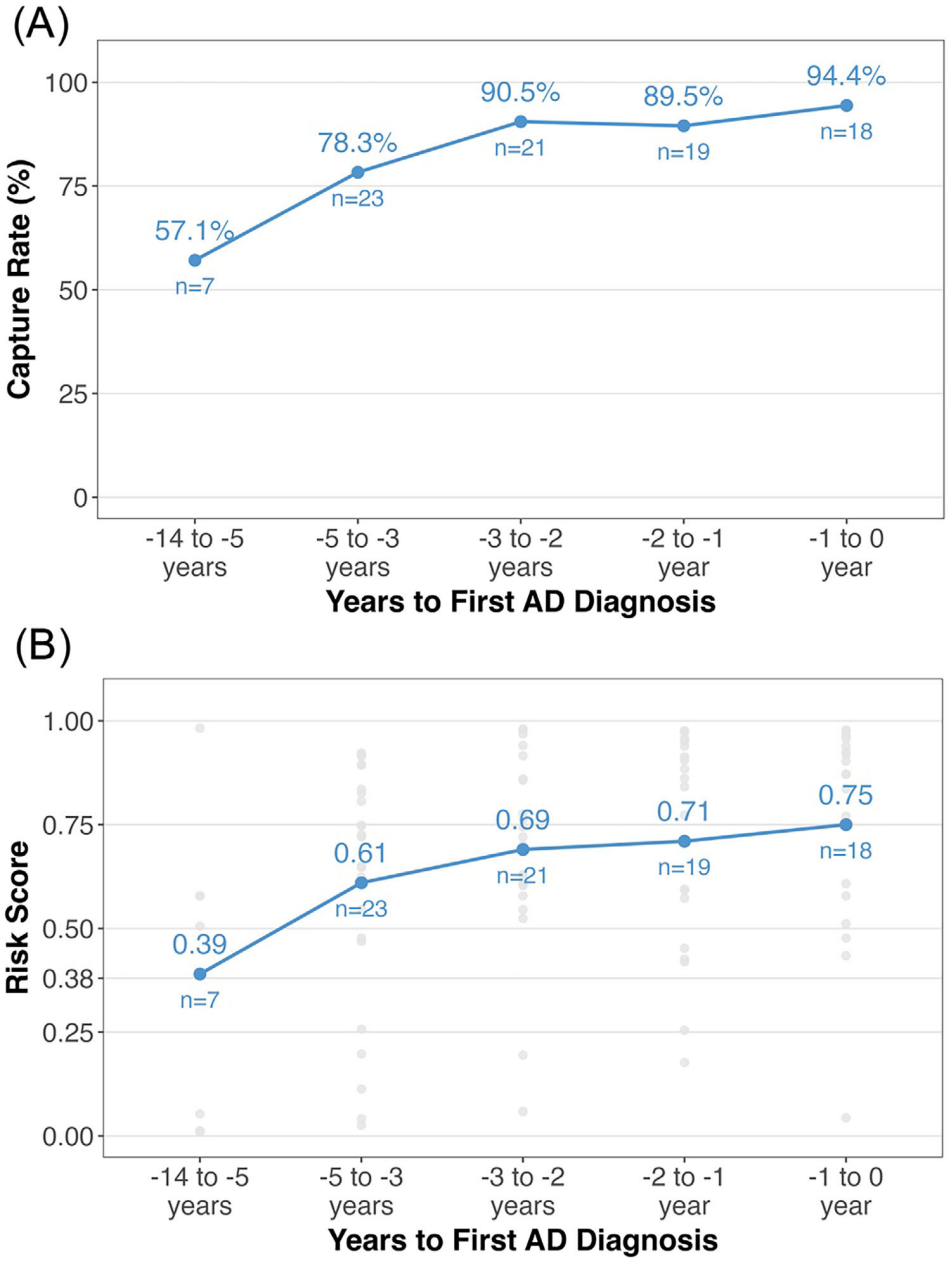
Proportion exceeding high‐risk criteria and mean sigmoid risk scores before the first AD diagnosis. The proportion of sessions with sigmoid risk scores meeting the high‐risk threshold (≥ 0.38) (A) and the mean sigmoid risk scores (B) across different time intervals before the first AD diagnosis. The numbers in (A) represent the proportions, while the numbers in (B) indicate the mean risk scores. *n* refers to the number of available sessions in each time period. The horizontal grey line at a risk score of 0.38 in (B) marks the cut‐off for high risk. AD, Alzheimer's disease.

### 3.6 Risk Scores Associated With Cognitive Assessments and Plasma Biomarkers

Linear mixed‐effects models were employed to examine the associations of MRI‐based AD risk scores with standardized cognitive assessments and plasma biomarker levels. Global associations were first assessed across all samples, without distinguishing diagnostic phases, to identify general patterns. Diagnosis‐specific analyses were then conducted within each pre‐AD diagnostic phase to evaluate more granular associations, given the importance of these phases for understanding early‐stage changes critical for detection and intervention.

As shown in Figure 5A and Table 2, MRI‐based risk scores were significantly associated with all cognitive measures across the entire dataset, including both global cognitive assessments (i.e., MMSE, MES, MOCA‐B) and subdomain‐specific tests (i.e., AVLT‐L, AVLT‐R, AFT, BNT, STT‐A, STT‐B). Higher MRI‐based risk scores were linked to poorer cognitive performance across the board. Specifically, higher scores on the STT‐A, and STT‐B (where higher scores indicate worse cognitive function) were associated with higher risk scores (*p*s < 0.01, *f^2^* s = 0.02). By contrast, for the remaining cognitive measures, where lower scores indicate worse cognitive function (e.g., MMSE, MES, MOCA‐B), higher risk scores were associated with lower scores, indicating a decline in cognitive performance (*p*s < 0.01, *f^2^* s = 0.02 – 0.10). When examined within each diagnostic phase, significant associations between MRI‐based risk scores and cognitive performance were observed only within the MCI phase (Figure 5B and Table 2). Specifically, risk scores remained significantly associated with MMSE (*p* < 0.001, *f^2^* = 0.04) and MOCA‐B (*p* < 0.05, *f^2^* = 0.05) within the MCI phase.

**FIGURE 5:**
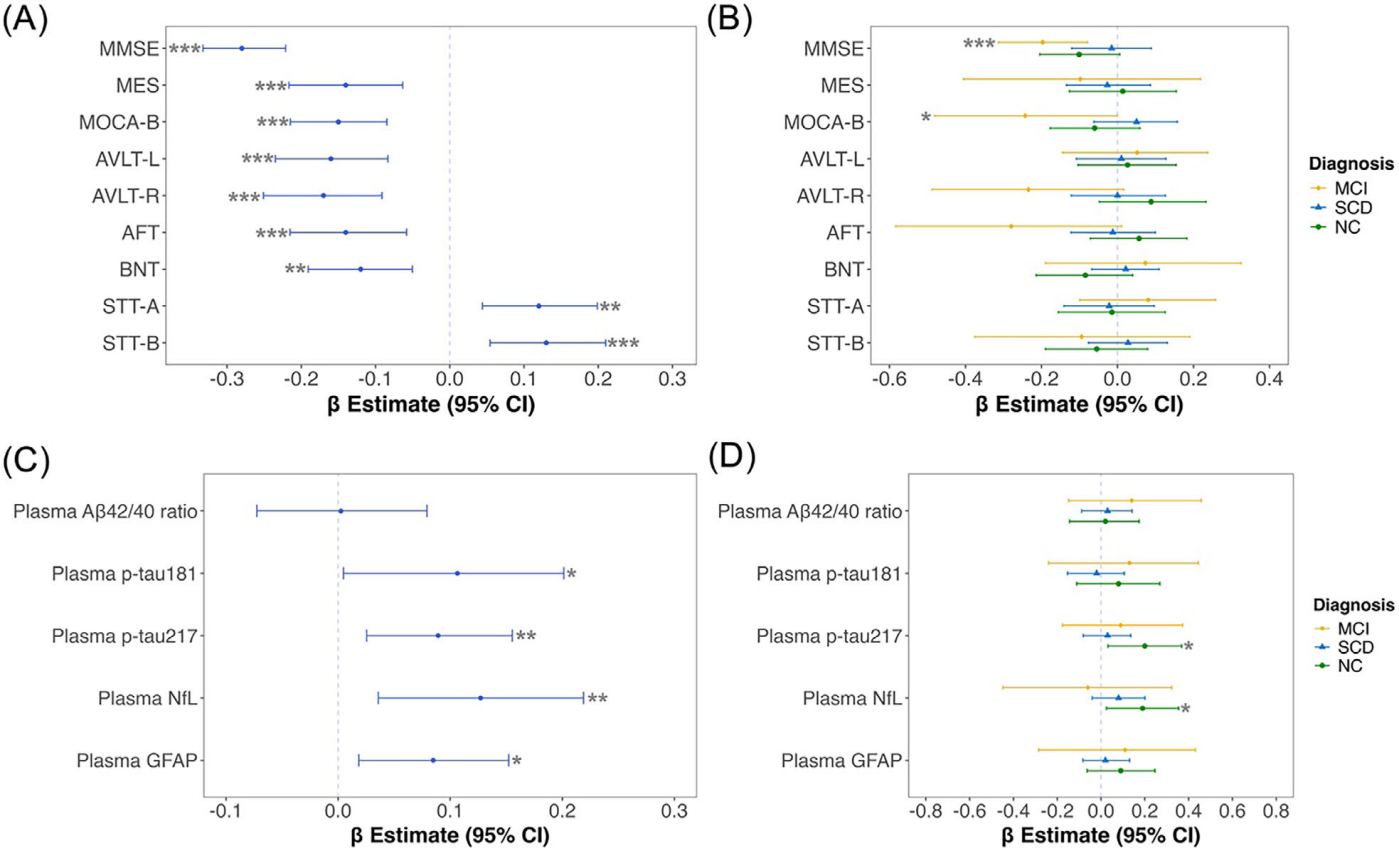
Risk score associations with cognitive performance and plasma biomarkers. (A,B) Associations between MRI‐based risk scores and cognitive performance across all samples (A) and within each pre‐AD group (B). (C,D) Associations between risk scores and plasma biomarker levels across all samples (C) and within each pre‐AD group (D). Separate models were fitted for each cognitive and plasma measure, with all variables standardized. The linear risk score was the independent variable, while sex, age, and education were included as covariates. Most models used linear mixed‐effects analysis, with participant included as a random effect. However, for plasma measures within the MCI group, general linear models without random effect were used instead due to the limited number of repeated measures per participant, which caused convergence issues in mixed‐effects models. Horizontal lines represent 95% confidence intervals (CIs; based on 5,000 bootstrap samples) for the *β* estimates of the linear risk score; CIs that do not include 0 indicate statistical significance. The dotted vertical line represents zero, with estimates to the left indicating negative associations and those to the right indicating positive associations. NC, normal cognition; SCD, subjective cognitive decline; MCI, mild cognitive impairment; AD, Alzheimer's disease; CI, confidence interval; MMSE, Mini‐Mental State Examination; MES, Memory and Executive Screening; MOCA‐B, Montreal Cognitive Assessment‐Basic; AVLT‐L, Auditory Verbal Learning Test—Long delayed memory; AVLT‐R, Auditory Verbal Learning Test— Recognition; AFT, Animal Fluency Test; BNT, Boston Naming Test; STT‐A, Shape Trail Test—Part A; STT‐B, Shape Trail Test—Part B; NfL, neurofilament light chain; GFAP, glial fibrillary acidic protein. ^*^ *p* < 0.05, ^**^ *p* < 0.01, ^***^ *p* < 0.001.

**TABLE 2:** Associations between MRI‐Based Risk Scores and Cognitive Measures and Plasma Biomarkers, across All Samples and by Diagnostic Phase.

	*All Samples*								*NC*							
*Measure*	*n*	*β*	*f^2^*	*SE*	*df*	*t*	*p*	95% *CI*	*n*	*β*	*f^2^*	*SE*	*df*	*t*	*p*	95% *CI*
**MMSE**	**1053**	−0.28***	0.10	0.03	1032.52	−9.97	2.03 × 10^−22^	[‐0.33, ‐0.22]	**330**	−0.1	0.01	0.05	320.2	−1.89	0.059	[‐0.2, 0.01]
**MES**	**492**	−0.14***	0.02	0.04	386.51	−3.63	3.27 × 10^−4^	[‐0.22, ‐0.06]	**188**	0.01	0	0.07	181.54	0.19	0.849	[‐0.13, 0.15]
**MOCA‐B**	**539**	−0.15***	0.03	0.03	373.46	−4.55	7.24 × 10^−6^	[‐0.21, ‐0.08]	**198**	−0.06	0	0.06	149.24	−1.00	0.321	[‐0.18, 0.06]
**AVLT‐L**	**540**	−0.16***	0.03	0.04	512.52	−4.07	5.38 × 10^−5^	[‐0.23, ‐0.08]	**198**	0.03	0	0.07	177.44	0.40	0.689	[‐0.1, 0.15]
**AVLT‐R**	**538**	−0.17***	0.03	0.04	512.44	−4.26	2.49 × 10^−5^	[‐0.25, ‐0.09]	**199**	0.09	0.01	0.07	180.21	1.23	0.221	[‐0.05, 0.23]
**AFT**	**509**	−0.14***	0.02	0.04	471.65	−3.37	8.14 × 10^−4^	[‐0.22, ‐0.06]	**191**	0.06	0	0.06	142.31	0.88	0.379	[‐0.07, 0.18]
**BNT**	**509**	−0.12**	0.02	0.04	378.24	−3.3	0.001	[‐0.19, ‐0.05]	**191**	−0.08	0.01	0.07	155.98	−1.30	0.195	[‐0.21, 0.04]
**STT‐A**	**506**	0.12**	0.02	0.04	459.61	3.01	0.003	[0.04, 0.20]	**191**	−0.01	0	0.07	179.16	−0.20	0.840	[‐0.16, 0.13]
**STT‐B**	**501**	0.13***	0.02	0.04	451.06	3.4	7.22 × 10^−4^	[0.05, 0.21]	**191**	−0.05	0	0.07	179.31	−0.80	0.423	[‐0.19, 0.08]
**Plasma Aβ42/40 ratio**	**377**	0.003	0	0.04	215.68	0.06	0.949	[‐0.07, 0.08]	**132**	0.02	0	0.08	74.89	0.22	0.824	[‐0.14, 0.17]
**Plasma p‐tau 181**	**361**	0.11*	0.01	0.05	328.24	2.22	0.027	[0.00, 0.20]	**124**	0.08	0.01	0.1	118.35	0.83	0.410	[‐0.11, 0.27]
**Plasma p‐tau 217**	**341**	0.09**	0.01	0.03	164.86	2.72	0.007	[0.03, 0.16]	**112**	0.2*	0.03	0.08	76	2.39	0.019	[0.03, 0.37]
**Plasma NfL**	**376**	0.13**	0.02	0.05	359.88	2.73	0.007	[0.04, 0.22]	**131**	0.19*	0.04	0.08	126	2.22	0.028	[0.02, 0.35]
**Plasma GFAP**	**379**	0.08*	0.01	0.03	198.03	2.47	0.014	[0.02, 0.15]	**132**	0.09	0	0.08	90.32	1.16	0.248	[‐0.06, 0.25]

	***SCD***								***MCI***							
*Measure*	*n*	*β*	*f^2^*	*SE*	*df*	*t*	*p*	95% *CI*	*n*	*β*	*f^2^*	*SE*	*df*	*t*	*p*	95% *CI*
**MMSE**	**337**	−0.02	0	0.05	311.05	−0.28	0.778	[‐0.12, 0.09]	**266**	−0.2***	0.04	0.06	259.94	−3.35	9.28× 10^−4^	[‐0.31, ‐0.08]
**MES**	**241**	−0.03	0	0.06	230.02	−0.47	0.642	[‐0.13, 0.09]	**49**	−0.1	0.01	0.15	29.94	−0.66	0.517	[‐0.41, 0.22]
**MOCA‐B**	**253**	0.05	0	0.05	246.27	0.91	0.363	[‐0.06, 0.16]	**67**	−0.24*	0.05	0.12	50.47	−2.05	0.046	[‐0.48, 0]
**AVLT‐L**	**254**	0.01	0	0.06	248.8	0.17	0.866	[‐0.11, 0.13]	**69**	0.05	0	0.09	24.39	0.56	0.582	[‐0.14, 0.24]
**AVLT‐R**	**253**	0	0	0.06	247.06	0	0.999	[‐0.12, 0.13]	**69**	−0.23	0.04	0.13	57.28	−1.87	0.067	[‐0.49, 0.02]
**AFT**	**245**	−0.01	0	0.06	231.12	−0.22	0.828	[‐0.12, 0.1]	**55**	−0.28	0.06	0.15	42.66	−1.92	0.061	[‐0.58, 0.01]
**BNT**	**245**	0.02	0	0.05	150.82	0.47	0.638	[‐0.07, 0.11]	**55**	0.07	0	0.13	47.97	0.57	0.574	[‐0.19, 0.33]
**STT‐A**	**245**	−0.02	0	0.06	238.83	−0.36	0.716	[‐0.14, 0.1]	**54**	0.08	0	0.09	11.49	0.91	0.382	[‐0.1, 0.26]
**STT‐B**	**244**	0.03	0	0.05	211.8	0.53	0.598	[‐0.08, 0.13]	**53**	−0.09	0.01	0.14	44.03	−0.67	0.503	[‐0.38, 0.19]
**Plasma Aβ42/40 ratio**	**199**	0.03	0	0.06	130.14	0.46	0.649	[‐0.09, 0.14]	**34**	0.14	0.02	0.17	29	0.85	0.404	[‐0.15, 0.46]
**Plasma p‐tau 181**	**194**	−0.02	0	0.07	188.23	−0.35	0.725	[‐0.15, 0.11]	**32**	0.13	0.02	0.19	27	0.72	0.476	[‐0.24, 0.45]
**Plasma p‐tau 217**	**184**	0.03	0	0.05	118.18	0.48	0.632	[‐0.08, 0.14]	**37**	0.09	0.01	0.14	32	0.64	0.528	[‐0.18, 0.37]
**Plasma NfL**	**202**	0.08	0.01	0.06	184.01	1.3	0.195	[‐0.04, 0.2]	**31**	−0.06	0	0.2	26	−0.28	0.783	[‐0.45, 0.32]
**Plasma GFAP**	**203**	0.02	0	0.05	140.09	0.44	0.659	[‐0.08, 0.13]	**32**	0.11	0.02	0.16	27	0.66	0.513	[‐0.28, 0.43]

Abbreviations: AFT, Animal Fluency Test; AVLT‐L, Auditory Verbal Learning Test—Long delayed memory; AVLT‐R, Auditory Verbal Learning Test— Recognition; BNT, Boston Naming Test; CI, confidence interval. MMSE, Mini‐Mental State Examination; df, degrees of freedom; GFAP, glial fibrillary acidic protein; MCI, mild cognitive impairment; MES, Memory and Executive Screening; MOCA‐B, Montreal Cognitive Assessment‐Basic;NC, normal cognition; NfL, neurofilament light chain; SCD, subjective cognitive decline; SE, standard error of the estimate; STT‐A, Shape Trail Test—Part A; STT‐B, Shape Trail Test—Part B.

NOTE: Associations of MRI‐based risk scores with cognitive measures and plasma biomarkers were examined across all samples and within each diagnostic group. *β* represents the regression coefficient of the linear risk score from each model, with all variables standardized for easier comparison. *f^2^* is Cohen's *f^2^*, a measure of effect size that indicates the extent to which the inclusion of a specific predictor variable improves the explanatory power of the model. All models adjusted for sex, age, and education. Most models included participants as a random effect, except for plasma measures within the MCI group, where general linear models without random effect were used to avoid convergence issues. 95% CI was computed using 5000 bootstrap iterations. ^*^ *p* < 0.05, ^**^ *p* < 0.01, ^***^ *p* < 0.001

When plasma biomarkers were examined across all samples (Figure 5C and Table 2), higher MRI‐based risk scores were positively associated with elevated levels of p‐tau 181, p‐tau 217, NfL, and GFAP (*p*s < 0.05, *f^2^* s = 0.01 – 0.02), indicating that higher risk scores are linked to increased levels of these neurodegenerative biomarkers. When examined by diagnosis, significant associations between MRI‐based risk scores and plasma biomarker levels were observed only within the NC phase (Figure 5D and Table 2). Specifically, risk scores remained significantly associated with p‐tau 217 (*p* < 0.05, *f^2^* = 0.03) and NfL (*p* < 0.05, *f^2^* = 0.04). In the SCD and MCI groups, the associations were all non‐significant (SCD: *p*s > 0.05, *f* ^2^s ≈ 0; MCI: *p*s > 0.05, *f* ^2^s = 0–0.02). The associations between risk scores and the plasma Aβ42/40 ratio were non‐significant, both across all samples and within diagnoses.

### 3.7 Subtyping and Predicting Cognitive Decline Speed via an Interpretability Method

Individualized AD brain risk maps were generated for each MRI session for all participants. In the AD group, all patients could be clustered into three categories based on the distribution of the risk areas, forming three MRI‐based AD subtypes (Figure S4). The current three‐cluster solution showed moderate‐to‐good cluster separation, with a mean silhouette width of 0.538 and a median silhouette width of 0.576, as well as high stability across 100 repeated k‐means initializations. The mean and median adjusted Rand indices (ARIs) relative to the best‐SSE solution were 0.854 and 0.920, respectively. Moreover, the current clustering solution showed high agreement with the best‐SSE solution (ARI = 0.920). The numbers of participants in the final clusters 1–3 were 14, 37, and 37, respectively. The essential high‐risk brain regions of the three AD subtypes are shown in Figures 6A–C. The primary risk regions for AD subtype 1 were located around the midbrain area, including the midbrain tectum, red nucleus, dorsal raphe nucleus, and pineal gland. The primary risk regions for AD subtype 2 included the hippocampus and posterior cingulate cortex. The primary risk regions for AD subtype 3 included the hippocampus, the midbrain tectum, red nucleus, dorsal raphe nucleus, and pineal gland. All SILCODE AD patients misclassified as NC by the MRI‐based classifier belong to AD subtype 1.

**FIGURE 6:**
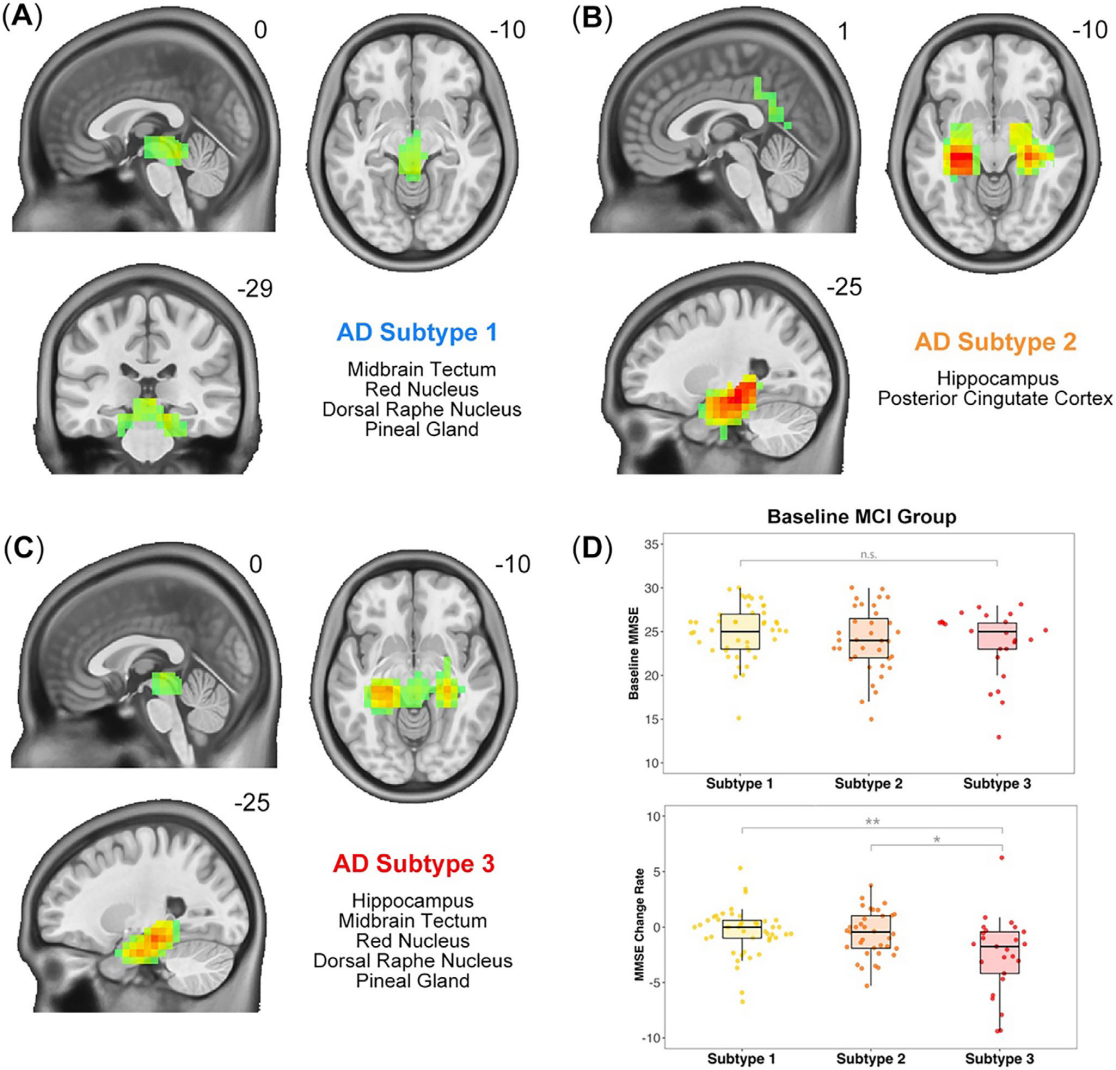
Alzheimer's disease subtypes defined by brain risk maps and their different rates of cognitive decline. (A–C) Distribution of brain regions with the highest disease risk for the three AD subtypes. The brain anatomical structures with the highest risks are listed in the bottom right of each of the panels. (D) Baseline MMSE levels and rates of decline across brain subtype groups of baseline MCI patients. AD, Alzheimer's disease; MMSE, Mini‐Mental State Examination; MCI, mild cognitive impairment. n.s., non‐significant, ^*^ *p* < 0.05, ^**^ *p* < 0.01.

Furthermore, results indicate that cognitive decline rates differed significantly among brain subtypes in the baseline MCI group, after adjusting for sex, age, and education (*F*(2, 105) = 3.63, *p* = 0.030; Figure 6D). Post‐hoc comparisons revealed that MCI patients classified as subtype 3 experienced a faster cognitive decline compared to those in subtype 1 (mean difference = 1.46, SE = 0.69, *t*(105) = 2.12, *p* = 0.036) and 2 (mean difference = 1.52, SE = 0.70, *t*(105) = 2.17, *p* = 0.032). In contrast, no significant differences were observed in baseline MMSE scores across the brain subtypes (*F*(2,105) = 1.56, *p* = 0.212; Figure 6D). Demographics and risk score profiles for different brain subtypes in the MCI group are presented in Figure S5. Cognitive decline rate did not differ significantly among subtypes in the NC or SCD groups (Figures S6 and S7).

## 4 Discussion

We examined the potential clinical utility of the MRI‐based AD deep learning model in a comprehensively phenotyped longitudinal Chinese cohort. The classifier, trained on North American samples, demonstrated good generalizability in distinguishing AD from NC within the Chinese sample. Moreover, the model accurately predicted disease progression in preclinical AD individuals, identifying 90.5% of individuals two to three years prior to their first clinical diagnosis. The MRI‐based risk scores were significantly correlated with global cognitive measurements and plasma‐based biomarkers, such as p‐tau 217. Finally, the interpretability method provided personalized brain risk maps and identified an MCI subtype that is prone to rapid cognitive decline. These predictive abilities enable the use of structural MRI to select patients who are most likely to benefit from closer monitoring and early intervention.

The classifier's ability to accurately distinguish AD from NC in the Chinese cohort and previously in the four Western samples underscores its robustness and cross‐population applicability [14]. Currently, MRI is primarily used for staging, monitoring disease progression, and detecting potential adverse events from pharmacological treatments [42]. However, MRI integrated with artificial intelligence is poised to be more closely integrated into screening, early diagnostics, and precision interventions for the AD spectrum [43, 44]. This may facilitate the decision‐making process for proceeding with monoclonal antibody treatments, thereby advancing intervention timing and effectiveness. In this study, the classifier's slightly lower performance than in the original study likely reflects differences in population characteristics. Among them, age might be most critical. The SILCODE cohort (66.6 ± 7.6 years) is significantly younger than the training data (76 ± 7 years) [14]. In fact, in the age‐matched SILCODE subset, the model achieved performance highly comparable to that reported in our previous study (Figure S3). Furthermore, according to the 2024 report by the Alzheimer's Association, AD incidence in the 75–84 age group is 2.64 times higher than in those aged 65–74 [45]. Considering the classifier's demonstrated ability in providing in‐advance alerts of AD progression risk, it is plausible that a considerable number of participants with high‐risk scores, currently diagnosed as NC, are likely to progress to AD in the future, warranting closer monitoring.

Notably, the model was trained primarily on North American non‐Latinx white participants [46]. Biological and cultural variations between ethnic groups can influence the cross‐population transferability of AD classifiers. For instance, studies have reported structural differences in brain gray matter volume and density between Caucasian and East Asian populations [47, 48]. Furthermore, the frequency of the APOE ε4 risk allele, one of the strongest genetic risk factors for AD, varies significantly between North American and Asian populations [49]. Sociocultural factors, including differences in social, physical, and dietary norms, may also impact the development of AD. Despite these potential confounders, the classifier achieved high accuracy in the Chinese cohort without any fine‐tuning. This performance is noteworthy, suggesting that the model captures core neuroanatomical AD markers that may generalize across ethnic and cultural contexts, supporting applicability in diverse populations. Moreover, the model's high sensitivity (95.2%) makes it well‐suited for AD screening and for selecting candidates for drug therapies, where minimizing missed high‐risk cases is the priority. Furthermore, although not trained on SCD or MCI patients, the model effectively distinguished different clinical stages, reflecting a continuous gradient of AD‐related pathology. Despite these strengths, the risk score should not be interpreted as a precise probability of AD without further recalibration.

Survival and lead time analyses further validated that MRI combined with deep learning could effectively predict early‐stage AD progression. In this study, the structural MRI‐based classifier was able to detect abnormality years before AD diagnosis, suggesting sensitivity to early neurodegenerative changes preceding substantial cognitive decline and visually observable atrophy [50, 51]. Moreover, the model‐derived risk score rises as diagnosis approaches, suggesting the classifier captures continuous prodromal neuroanatomical progression. Furthermore, MCI individuals identified as high‐risk typically exhibit rapid disease progression, as indicated by a substantially short time to conversion (i.e., 31 months), highlighting the urgent need for timely intervention. Cox proportional hazards regression further confirmed this prognostic value, as higher baseline risk scores were significantly associated with an increased hazard of future conversion to AD, with this association remaining significant after adjustment for demographic and diagnostic factors. Currently, the emerging treatments targeting amyloid plaques and tau pathology are more effective when applied during the earliest phases of neurodegeneration [52, 53]. The classifier was able to identify 90.5% of high‐risk individuals two to three years prior to progression, thereby opening a time window for timely pharmacological intervention, potentially delaying AD progression and improving long‐term outcomes. However, the results for pre‐diagnosis intervals exceeding 5 years should be interpreted with caution because of the limited sample size.

The individualized brain risk maps offer a novel approach to understand and visualize disease pathology and progression at the individual level. This approach may facilitate targeted interventions and enhance physician confidence in the deep learning model. Interestingly, the AD‐related risk areas include not only the heavily affected hippocampus and posterior cingulate cortex, but also deep structures such as the midbrain tectum, red nucleus, dorsal raphe nucleus, and pineal gland, which are particularly pronounced in subtype 1. A substantial amount of morphological and pathological evidence shows that these structures are altered in AD and closely linked to symptoms like sleep disturbances, impaired audiovisual integration, and depressed mood [54, 55, 56, 57, 58]. For example, the deposition of the RNA‐binding protein TDP‐43 in the midbrain tectum is considered a key feature of Stage 5 in the latest AD staging scheme [59, 60]. Additionally, the pineal gland in AD patients is significantly smaller than in NC and MCI, and its volume is associated with cognitive decline [58]. Subtype 3 demonstrated a significantly accelerated rate of cognitive decline. Notably, the three subgroups exhibited comparable baseline MMSE performance, underscoring the incremental prognostic and stratification value of brain risk maps beyond traditional MMSE measures. Considering their more rapid cognitive deterioration, MCI individuals classified as subtype 3 may warrant intensified follow‐up and monitoring. Finally, individualized brain risk maps may provide a method for identifying candidate targets for non‐invasive neuromodulation by delineating the brain regions most strongly implicated in elevated AD risk.

Moving beyond the CSF‐based approaches of prior studies [61, 62], our research evaluates the physiological relevance of an MRI‐derived risk score using plasma biomarkers. Compared to CSF biomarkers, blood‐based biomarkers provide a less invasive, more accessible, and potentially more scalable diagnostic solution. For this evaluation, we employed the A‐T‐N‐I framework: Amyloid (Aβ 42/40 ratio), Tau (p‐tau 181 and p‐tau 217), Neurodegeneration (NfL), and Inflammation (GFAP). Among blood biomarkers, the risk score showed significant global correlations with T, N, and I, indicating concordance between MRI‐derived structural alterations and plasma biochemical changes. Within diagnostic groups, significant associations emerged only in the NC group, consistent with the notion that plasma biomarkers change most rapidly during the preclinical stage [63]. Notably, the risk score was significantly associated with plasma p‐tau217, the most reliable phosphorylated tau biomarker for distinguishing preclinical pathological stages [64] and predicting progression [65], but not with p‐tau181. Importantly, in the 2024 revision of the NIA‐AA criteria, p‐tau 217 is the sole plasma biomarker utilized for diagnostic purposes and the only plasma biomarker that is used simultaneously for diagnosis, staging, monitoring progression, and assessing treatment efficacy [17]. Besides, NfL levels show concurrent alterations with brain anatomy along the AD progression trajectory [4, 5] and predict hippocampal volume changes—a key feature of AD pathology—in cognitively unimpaired individuals [66]. Notably, similar associations in MCI cannot be excluded, as the small MCI subset with both plasma and MRI data (∼30) limited statistical power. The availability of each cognitive and plasma measure across diagnostic groups is detailed in Table 1. Analyses of associations with cognitive and plasma measures included only participants with the required data, and no imputation or other missing‐data methods were used. Interestingly, the amyloid biomarker did not significantly correlate with model scores. Supporting this, Table 1 shows nearly identical plasma Aβ42/40 ratios across diagnoses. Prior studies similarly found no significant differences across AD stages and only weak correlations between plasma Aβ42/40 and its CSF or PET counterparts [67, 68]. Measurement methods also affect results. A comparative study found that Simoa immunoassays, particularly from Quanterix, as used in the current dataset, do not adequately represent CSF Aβ42/40 or Aβ PET status [69].

Finally, associations of MRI‐based risk scores with both global and subdomain‐specific cognitive measures were significant across the whole sample. Within diagnosis, significant associations were observed only during the MCI phase, where cognitive symptoms deteriorate significantly [63]. The remaining significant associations of risk scores with MMSE and MOCA‐B, two widely used global cognitive measures, support the clinical applicability of MRI‐based risk.

Study limitations include the use of a single‐site dataset, which hinders effective simultaneous fine‐tuning and testing of the model. Future work should leverage larger, more representative multicenter AD datasets to fine‐tune the model, improve generalizability, and enhance clinical utility. Besides, due to the retrospective nature of the brain risk mapping approach, the highlighted risky regions may be sufficient but not necessary for reflecting AD pathology. Other regions may also harbor pathological abnormalities but remain undetected by post‐hoc interpretability techniques, indicating the need for developing new analytical approaches. Missingness in cognitive assessments and plasma biomarker measurements should be acknowledged. Missing data may be related to factors such as participant compliance, disease severity, and loss to follow‐up, and the resulting potential selection bias should therefore be considered when interpreting these associations.

In summary, the MRI‐based deep learning AD classifier generalized well to an age‐ and ethnically distinct population in AD classification and progression prediction. Integrating MRI into clinical practice can complement current biomarkers, facilitating the selection of patients most likely to benefit from prospective monitoring and early immunotherapy interventions, thereby supporting clinical decision‐making and precision medicine.

## Author Contributions

**C‐GY**: conceptualized and designed the research, interpreted results, revised the manuscript, and supervised the entire research process. **YH**: conceptualized the research, interpreted results, acquired the data, and provided expert domain knowledge in the AD research field. **JL**: interpreted results, acquired the data, and provided expert domain knowledge in the AD research field. **BL**: conceptualized and designed the research, conducted data analysis and visualization, interpreted results, and drafted the manuscript. **Y‐RC**: designed the evaluation framework, conducted data analysis and visualization, interpreted results, and drafted the manuscript. **R‐XL, M‐KZ, S‐ZY, G‐QC, J‐HW, W‐HC**: acquired data and provided diagnostic and data collection details. **FXC** and **PMT**: revised the manuscript.

## Funding

Funding for the study was provided by the Brain Science and Brain‐like Intelligence Technology‐National Science and Technology Major Project (2022ZD0211800 [YH], 2021ZD0200600 [C‐GY]), National Natural Science Foundation of China (82572192 [C‐GY], 82327809 [YH]), the Construction Fund of Key Medical Disciplines of Hangzhou (2025HZGF02 [YH]), Scientific Foundation of Institute of Psychology, Chinese Academy of Sciences (E3CX1425CX [BL]), the Qiantang Scholars Program (YH), Beijing Nova Program of Science and Technology (20230484465 [C‐GY]), Beijing Natural Science Foundation (J230040 [C‐GY]). None of the funding sources had any involvement in the study design, data analysis, or manuscript writing.

## Consent Statement

All human subjects provided informed consent.

## Conflicts of Interest

The authors declare no conflicts of interest.

## Supporting information




**Supporting File**: advs78537‐sup‐0001‐SuppMat.docx.

## Data Availability

The SILCODE dataset used in this study is not publicly available due to participant privacy and ethical considerations. However, researchers interested in collaboration may contact the corresponding authors to inquire about collaboration opportunities and apply for data access. Applicants are required to provide a detailed research plan and sign a data use agreement. The research team will respond within 30 business days upon receiving applications. All data sharing will strictly adhere to relevant privacy protections and ethical guidelines to ensure the secure and compliant use of the data. The code for the MRI‐based deep learning model for Alzheimer's disease is available at https://github.com/Chaogan‐Yan/BrainImageNet. Our demonstration website (http://brainimagenet.org) allows users to upload data directly and obtain risk scores after providing informed consent. Additionally, the codes for statistical analyses, creating individualized AD brain risk maps, and data visualization are available at https://github.com/Chaogan‐Yan/BrainImageNet/tree/master/ADPredictionPaperScripts.

## Footnotes

1 Model scores derived from linear activation, which are better suited for statistical testing than the default sigmoid‐activated risk scores. See Methods for further details.
