# Supplementary material for: Deep Learning for AD Risk Screening and Progression Prediction
Source: Adv Sci (Weinh). 2026 Nov 5:e78537. Online ahead of print. doi: 10.1002/advs.78537 (PMC13735437; doi:10.1002/advs.78537)
Supplement: Supplementary file 1 [file ADVS-9999-e78537-s001.docx]

Supplemental Materials for

**Deep Learning for AD Risk Screening and Progression Prediction**

Bin Lu^#^, Yan-Rong Chen^#^, Rui-Xian Li^#^, Ming-Kai Zhang, Shao-Zhen Yan, Guan-Qun Chen, Jing-Hua Wang, Wen-Hui Chai, Francisco Xavier Castellanos, Paul M. Thompson, Jie Lu*, Ying Han*, Chao-Gan Yan*

\# These authors contributed equally as the first author.

*Corresponding authors:

Chao-Gan Yan, Ph.D. Address: Room 304, Weiqing Building, Tsinghua University, Haidian District, Beijing, 100084. E-mail: yancg@tsinghua.edu.cn Telephone: (+86) 131-4614-8848.

Ying Han, Ph.D., Address: 45 Changchun Street, Xicheng District, Beijing 100053, China. E-mail: hanying@xwh.ccmu.edu.cn, Telephone: (+86) 136-2101-1941.

Jie Lu, Ph.D. Address: 45 Changchun Street, Xicheng District, Beijing 100053, China. E-mail: imaginglu@hotmail.com, Telephone: (+86) 189-1150-7070.

Table of Contents

[Supplementary Methods	1](#_Toc238897312)

[S1. Subject-level analysis	1](#_Toc238897313)

[S2. Age-matched analysis	1](#_Toc238897314)

[S3. Interaction analyses of MRI-based AD risk scores with diagnosis stage and demographic characteristics for cognitive and plasma biomarker outcomes	1](#_Toc238897315)

[Supplementary Figures	3](#_Toc238897316)

[FIGURE S1 Frequency distribution of intervals between MRI session dates and dates of progression to Alzheimer’s disease (AD) in people who converted to AD during the follow-up period.	3](#_Toc238897317)

[FIGURE S2 Subject-level classification performance of the MRI-based AD classifier on the SILCODE Cohort.	4](#_Toc238897318)

[FIGURE S3 Receiver operating characteristic (ROC) analysis for session-level AD vs. NC classification in SILCODE subsamples age-matched to the Lu et al. (2022) training cohort.	6](#_Toc238897319)

[FIGURE S4 Determination of the optimal number of clusters for k-means clustering.	7](#_Toc238897320)

[FIGURE S5 Demographic and risk score profiles across different subtype patterns within baseline MCI group.	8](#_Toc238897321)

[FIGURE S6 Baseline scores and change rates of MMSE scores across different subtype patterns within baseline NC group.	9](#_Toc238897322)

[FIGURE S7 Baseline scores and change rates of MMSE scores across different subtype patterns within baseline SCD group.	10](#_Toc238897323)

[FIGURE S8 Calibration Plot of the Risk Score	11](#_Toc238897324)

[Supplementary Tables	12](#_Toc238897325)

[Table S1 Classification performance at the SILCODE-derived Youden threshold of 0.38 and the default threshold of 0.50.	12](#_Toc238897326)

[Table S2. Moderating effects of sex, age, education, and baseline diagnostic stage on associations between MRI-based AD risk scores and cognitive and plasma biomarker outcomes	13](#_Toc238897327)

[Table S3. Follow-up analyses of significant interactions between MRI-based AD risk scores and sex, age, education, and diagnostic stage for cognitive and plasma biomarker outcomes	14](#_Toc238897328)

# Supplementary Methods

## S1. Subject-level analysis

As a supplementary analysis conducted in response to concerns about subject-weighting bias arising from participants with multiple visits, we conducted a supplementary subject-level analysis for AD–NC classification and for comparisons of risk scores across clinical diagnoses. For this analysis, a single index scan was selected for each participant. Specifically, the last available MRI scan for each individual was used as the representative scan to ensure a balanced representation of disease stage across diagnostic groups. This yielded a subsample consisting of 218 NC participants and 117 AD patients. The corresponding results are presented in Figure S2.

## S2. Age-matched analysis

To evaluate the potential impact of age distribution differences between the SILCODE cohort and the original training cohort (Lu et al., 2022), a supplementary sensitivity analysis was conducted using an age-matched subset. The original training cohort consisted of AD patients with a mean age of 75.97 (SD = 7.85) and NC participants with a mean age of 76.08 (SD = 6.53). To construct comparable subsets from the SILCODE cohort, AD and NC participants were matched separately to the corresponding age distributions in the original training cohort. Specifically, within each diagnostic group, we used an iterative random subsampling procedure to identify a subset whose mean age and age variability were statistically comparable to those of the corresponding training group. For each candidate subset size, random sampling without replacement was repeated for up to 50,000 iterations. For each randomly selected subset, a one-sample t-test was used to assess whether its mean age differed from the target mean, and a chi-square test of variance was used to assess whether its age variance differed from the target variance. A subset was considered successfully matched when both tests yielded *p* > 0.05. The procedure started with the largest possible subset and progressively reduced the subset size until a subset meeting both criteria was identified. This process identified a SILCODE subsample consisting of 76 AD scans (mean age 74.34, SD 8.72) and 12 NC scans (mean age 71.25, SD 7.75). Statistical analyses confirmed that there were no significant differences in mean age or standard deviation between this matched subsample and the original training cohort for either group (all *p*s > 0.05). Classification results for this age-matched analysis are presented in Figure S3.

## S3. Interaction analyses of MRI-based AD risk scores with diagnosis stage and demographic characteristics for cognitive and plasma biomarker outcomes

To evaluate whether associations between the MRI-based AD risk score and cognitive or plasma biomarker outcomes varied by diagnosis stage or demographic characteristics, formal interaction analyses were conducted separately for each outcome. For each outcome, a linear mixed-effects model was fitted with the risk score, diagnosis stage, age, sex, and education as main effects, together with their corresponding interactions with the risk score:

Outcome=Risk+Diagnosis+Age+Sex+Education+Risk×Diagnosis+Risk×Sex+Risk×Age+Risk×Education+(1∣Participant).

All continuous variables, including the risk score, age, education, and outcome measures, were standardized before entering models. Sex and diagnosis stage were treated as categorical variables and sum-coded. Type III Wald χ² tests were used to evaluate the statistical significance of the interaction terms. When a significant interaction was detected, follow-up analyses were conducted to characterize the nature and direction of the interaction. For continuous moderators (age and education), simple slopes of the risk score were estimated at −1 SD, the mean, and +1 SD of the moderator. For categorical moderators (sex and diagnosis), risk-score slopes were estimated within each group, followed by Tukey-adjusted pairwise comparisons of the slopes.

# Supplementary Figures

## FIGURE S1 Frequency distribution of intervals between MRI session dates and dates of progression to Alzheimer’s disease (AD) in people who converted to AD during the follow-up period.

## FIGURE S2 Subject-level classification performance of the MRI-based AD classifier on the SILCODE Cohort.

(A) Receiver operating characteristic (ROC) analysis for subject-level classification of AD vs. NC. The solid blue line represents the mean ROC curve, and the light blue shaded area indicates the 95% confidence interval (CI). The classifier achieved an area under the curve (AUC) of 0.909 (95% CI: 0.876–0.940). An optimal cutoff score of 0.38 was identified using the Youden index, which is identical to the cutoff obtained using session-level data. At this threshold, the classifier achieved an accuracy of 88.4% (95% CI: 84.4 – 91.6%), a sensitivity of 95.7% (95% CI: 90.3 – 98.6%), a specificity of 84.4% (95% CI: 78.9 – 89.0%), a positive predictive value (PPV) of 76.7% (95% CI: 69.0 – 83.3%), and a negative predictive value (NPV) of 97.4% (95% CI: 93.9 – 99.1%). (B) Risk scores across diagnostic groups at the subject level. Box plots show the median, interquartile range, and whiskers extending 1.5 times the interquartile range, with individual data points displayed. For subject-level analysis, after adjusting for sex, age, and education, we found that linear risk scores varied significantly by clinical diagnoses (F(3, 715) = 74.77, p < 2.20 × 10-16). Post-hoc Tukey-corrected comparisons revealed that the AD group had significantly higher scores than NC (mean difference = 3.65, SE = 0.25, t(715) = 14.44, p < 2.20 × 10-16), SCD (mean difference = 2.64, SE = 0.25, t(715) = 10.44, p < 2.20 × 10-16), and MCI groups (mean difference = 1.61, SE = 0.26, t(715) = 6.26, p = 3.59 × 10-10). MCI also scored higher than NC (mean difference = 2.04, SE = 0.22, t(715) = 9.14, p < 2.20 × 10-16) and SCD (mean difference = 1.03, SE = 0.23, t(715) = 4.57, p = 3.40× 10-5). In addition, SCD scored higher than NC (mean difference = 1.01, SE = 0.20, t(715) = 4.97, p = 4.89× 10-6). ***p < 0.001

## FIGURE S3 Receiver operating characteristic (ROC) analysis for session-level AD vs. NC classification in SILCODE subsamples age-matched to the Lu et al. (2022) training cohort.

The solid blue line represents the ROC curve, and the light blue shaded area indicates the 95% confidence interval (CI). The classifier achieved an area under the curve (AUC) of 0.962 (95% CI: 0.917–0.992). An optimal cutoff score of 0.49 was identified using the Youden index. At this threshold, the classifier achieved an accuracy of 86.4% (95% CI: 77.4–92.8%), a sensitivity of 84.2% (95% CI: 74.0–91.6%), and a specificity of 100.0% (95% CI: 73.5–100.0%). The PPV was 100.0% (95% CI: 94.4–100.0) and the NPV was 50.0% (95% CI: 29.1–70.9%). These estimates should be interpreted cautiously given the small number of NC scans in the age-matched subset.

## FIGURE S4 Determination of the optimal number of clusters for k-means clustering.

(A) Elbow plot based on the sum of squared errors (SSE). (B) Mean silhouette width across different numbers of clusters.

## FIGURE S5 Demographic and risk score profiles across different subtype patterns within baseline MCI group.

(A) Age. (B) Sex Percentage. (C) Years of Education. (D) Risk Score. MCI, mild cognitive impairment. n.s., non-significant, **p < .01, ***p < .001.

## FIGURE S6 Baseline scores and change rates of MMSE scores across different subtype patterns within baseline NC group.

(A) Baseline MMSE scores. (B) MMSE change rates. MMSE, Mini-Mental State Examination; NC, normal cognition; n.s., non-significant.

## FIGURE S7 Baseline scores and change rates of MMSE scores across different subtype patterns within baseline SCD group.

(A) Baseline MMSE scores. (B) MMSE change rates. MMSE, Mini-Mental State Examination; SCD, subjective cognitive decline; n.s., non-significant.

## FIGURE S8 Calibration Plot of the Risk Score

The calibration plot shows the observed proportion of AD cases within deciles of the predicted risk score, plotted against the mean predicted risk in each decile. The red diagonal line represents perfect calibration, the black line represents logistic calibration, and the gray curve represents flexible calibration estimated using Loess, with gray dashed lines indicating its 95% confidence interval. Triangles represent grouped observations, with vertical error bars indicating 95% confidence intervals calculated using exact binomial methods. The calibration intercept was −0.74, the calibration slope was 0.96, and the Brier score was 0.11. The flexible calibration curve showed varying degrees and directions of deviation from perfect calibration across the score range.

# Supplementary Tables

## Table S1 Classification performance at the SILCODE-derived Youden threshold of 0.38 and the default threshold of 0.50.

| Performance Metric | Threshold = 0.38 | Threshold = 0.50 |
| --- | --- | --- |
| Accuracy (95% CI) | 88.2% (84.9–90.9%) | 86.5% (83.1–89.4%) |
| Sensitivity (95% CI) | 95.2% (89.8–98.2%) | 82.4% (74.6–88.6%) |
| Specificity (95% CI) | 85.6% (81.5–89.1%) | 87.9% (84.0–91.2%) |
| PPV (95% CI) | 70.4% (62.9–77.2%) | 71.0% (62.9–78.3%) |
| NPV (95% CI) | 98.0% (95.8–99.3%) | 93.3% (90.0–95.7%) |

Abbreviations: PPV, positive predictive value; NPV, negative predictive value.

NOTE: The 95% confidence intervals were calculated using exact binomial methods. Classification performance was broadly similar across the two thresholds. Compared with the default threshold of 0.50, the lower threshold of 0.38 resulted in a slight decrease in specificity (87.9% vs. 85.6%) and PPV (71.0% vs. 70.4%), a slight increase in accuracy (86.5% vs. 88.2%), and higher sensitivity (82.4% vs. 95.2%) and NPV (93.3% vs. 98.0%)

## Table S2. Moderating effects of sex, age, education, and baseline diagnostic stage on associations between MRI-based AD risk scores and cognitive and plasma biomarker outcomes

| **Outcome** | **Risk × Sex χ² (df)** | ***p*** | **Risk × Age χ² (df)** | ***p*** | **Risk × Education χ² (df)** | ***p*** | **Risk × Diagnosis χ² (df)** | ***p*** |
| --- | --- | --- | --- | --- | --- | --- | --- | --- |
| MMSE | 0.00 (1) | .997 | **5.18 (1)** | **.023** | 0.00 (1) | .952 | **19.29 (3)** | **<.001** |
| MES | 3.61 (1) | .058 | 2.05 (1) | .152 | 1.06 (1) | .304 | **36.37 (3)** | **<.001** |
| MOCA-B | 0.08 (1) | .774 | 0.79 (1) | .374 | 1.77 (1) | .183 | **16.27 (3)** | **<.001** |
| AVLT-L | 1.91 (1) | .167 | 0.05 (1) | .822 | 3.84 (1) | .050 | 1.51 (3) | .680 |
| AVLT-R | 1.53 (1) | .217 | 0.56 (1) | .454 | 0.29 (1) | .588 | **9.81 (3)** | **.020** |
| AFT | 0.49 (1) | .484 | 2.24 (1) | .135 | 0.01 (1) | .945 | 6.22 (3) | .101 |
| BNT | 0.23 (1) | .634 | 2.86 (1) | .091 | 0.73 (1) | .394 | **18.75 (3)** | **<.001** |
| STT-A | 1.63 (1) | .202 | **17.40 (1)** | **<.001** | 0.50 (1) | .480 | 6.39 (3) | .094 |
| STT-B | 0.36 (1) | .548 | 0.03 (1) | .865 | 0.38 (1) | .538 | 7.81 (3) | .050 |
| **Plasma Aβ42/40** | **4.19 (1)** | **.041** | 0.05 (1) | .826 | 0.28 (1) | .595 | 1.38 (3) | .710 |
| Plasma p-tau181 | 2.48 (1) | .115 | 0.37 (1) | .545 | 0.64 (1) | .424 | 1.81 (3) | .613 |
| Plasma p-tau217 | 0.29 (1) | .591 | 0.25 (1) | .617 | 2.33 (1) | .127 | 3.94 (3) | .269 |
| Plasma NfL | 0.35 (1) | .553 | 2.79 (1) | .095 | 1.75 (1) | .186 | 3.15 (3) | .370 |
| Plasma GFAP | 0.00 (1) | .968 | 0.62 (1) | .433 | 0.36 (1) | .547 | 3.57 (3) | .312 |

NOTE: Type III Wald χ² tests were used to evaluate interactions between the standardized continuous MRI-based AD risk score and sex, age, education, and baseline diagnostic stage. All continuous variables, including risk scores, age, education, and outcome measures, were standardized; sex and baseline diagnosis were sum-coded. Models included the main effects of risk score and moderators, their interaction terms, and a random intercept for participant. Significant interactions were followed by simple-slope analyses or Tukey-adjusted pairwise comparisons, as appropriate (see Table S3). *p* values < .05 are shown in bold.

## Table S3. Follow-up analyses of significant interactions between MRI-based AD risk scores and sex, age, education, and diagnostic stage for cognitive and plasma biomarker outcomes

| Outcome | Moderator | Simple Slope/  Comparison | Estimate | SE | df | CI | Adjusted_p |
| --- | --- | --- | --- | --- | --- | --- | --- |
| MMSE | Risk × Diagnosis | NC | -0.018 | 0.04 | 987.05 | [-0.097, 0.062] |  |
| MMSE | Risk × Diagnosis | SCD | 0.011 | 0.04 | 1030.2 | [-0.064, 0.086] |  |
| MMSE | Risk × Diagnosis | MCI | -0.167 | 0.04 | 1037.6 | [-0.245, -0.09] |  |
| MMSE | Risk × Diagnosis | AD | -0.266 | 0.07 | 1029.9 | [-0.4, -0.132] |  |
| MMSE | Risk × Diagnosis | NC - SCD | -0.029 | 0.05 | 976.83 |  | 0.949 |
| **MMSE** | **Risk × Diagnosis** | **NC - MCI** | **0.149** | **0.06** | **1018.5** |  | **0.037** |
| **MMSE** | **Risk × Diagnosis** | **NC - AD** | **0.249** | **0.08** | **1024.8** |  | **0.011** |
| **MMSE** | **Risk × Diagnosis** | **SCD - MCI** | **0.178** | **0.06** | **1036.9** |  | **0.007** |
| **MMSE** | **Risk × Diagnosis** | **SCD - AD** | **0.277** | **0.08** | **1032.8** |  | **0.003** |
| MMSE | Risk × Diagnosis | MCI - AD | 0.099 | 0.08 | 1025.4 |  | 0.587 |
| **MMSE** | **Risk × Age** | **−1 SD** | **-0.153** | **0.03** | **1028.8** | **[-0.212, -0.095]** |  |
| **MMSE** | **Risk × Age** | **Mean** | **-0.11** | **0.03** | **1031.6** | **[-0.158, -0.062]** |  |
| **MMSE** | **Risk × Age** | **+1 SD** | **-0.067** | **0.03** | **1025.1** | **[-0.13, -0.003]** |  |
| MES | Risk × Diagnosis | NC | 0.041 | 0.06 | 431.31 | [-0.07, 0.152] |  |
| MES | Risk × Diagnosis | SCD | 0.017 | 0.04 | 450.56 | [-0.069, 0.103] |  |
| MES | Risk × Diagnosis | MCI | -0.064 | 0.09 | 478 | [-0.236, 0.107] |  |
| MES | Risk × Diagnosis | AD | -1.077 | 0.18 | 414.95 | [-1.436, -0.717] |  |
| MES | Risk × Diagnosis | NC - SCD | 0.024 | 0.07 | 418.13 |  | 0.985 |
| MES | Risk × Diagnosis | NC - MCI | 0.105 | 0.1 | 472.69 |  | 0.741 |
| **MES** | **Risk × Diagnosis** | **NC - AD** | **1.118** | **0.19** | **432.57** |  | **<.001** |
| MES | Risk × Diagnosis | SCD - MCI | 0.081 | 0.1 | 474.86 |  | 0.836 |
| **MES** | **Risk × Diagnosis** | **SCD - AD** | **1.094** | **0.19** | **426.71** |  | **<.001** |
| **MES** | **Risk × Diagnosis** | **MCI - AD** | **1.012** | **0.21** | **416.63** |  | **<.001** |
| MOCA-B | Risk × Diagnosis | NC | -0.041 | 0.05 | 451.97 | [-0.143, 0.06] |  |
| MOCA-B | Risk × Diagnosis | SCD | 0.025 | 0.04 | 499.85 | [-0.054, 0.104] |  |
| MOCA-B | Risk × Diagnosis | MCI | -0.259 | 0.07 | 524.9 | [-0.401, -0.117] |  |
| MOCA-B | Risk × Diagnosis | AD | -0.339 | 0.15 | 524.51 | [-0.632, -0.047] |  |
| MOCA-B | Risk × Diagnosis | NC - SCD | -0.067 | 0.06 | 451.52 |  | 0.709 |
| MOCA-B | Risk × Diagnosis | NC - MCI | 0.218 | 0.09 | 516.05 |  | 0.070 |
| MOCA-B | Risk × Diagnosis | NC - AD | 0.298 | 0.16 | 524.89 |  | 0.232 |
| **MOCA-B** | **Risk × Diagnosis** | **SCD - MCI** | **0.284** | **0.08** | **522.61** |  | **0.004** |
| MOCA-B | Risk × Diagnosis | SCD - AD | 0.365 | 0.15 | 524.96 |  | 0.082 |
| MOCA-B | Risk × Diagnosis | MCI - AD | 0.081 | 0.17 | 520.95 |  | 0.963 |
| AVLT-R | Risk × Diagnosis | NC | 0.069 | 0.07 | 519.06 | [-0.066, 0.204] |  |
| AVLT-R | Risk × Diagnosis | SCD | 0.019 | 0.05 | 520.55 | [-0.082, 0.121] |  |
| AVLT-R | Risk × Diagnosis | MCI | -0.259 | 0.09 | 521.11 | [-0.434, -0.084] |  |
| AVLT-R | Risk × Diagnosis | AD | -0.139 | 0.2 | 519.97 | [-0.537, 0.259] |  |
| AVLT-R | Risk × Diagnosis | NC - SCD | 0.05 | 0.08 | 520.52 |  | 0.933 |
| **AVLT-R** | **Risk × Diagnosis** | **NC - MCI** | **0.328** | **0.11** | **524** |  | **0.019** |
| AVLT-R | Risk × Diagnosis | NC - AD | 0.208 | 0.21 | 521.63 |  | 0.765 |
| **AVLT-R** | **Risk × Diagnosis** | **SCD - MCI** | **0.278** | **0.1** | **522.05** |  | **0.037** |
| AVLT-R | Risk × Diagnosis | SCD - AD | 0.158 | 0.21 | 520.72 |  | 0.872 |
| AVLT-R | Risk × Diagnosis | MCI - AD | -0.12 | 0.22 | 517.67 |  | 0.950 |
| BNT | Risk × Diagnosis | NC | -0.065 | 0.06 | 382.59 | [-0.189, 0.059] |  |
| BNT | Risk × Diagnosis | SCD | 0.006 | 0.05 | 405.47 | [-0.091, 0.103] |  |
| BNT | Risk × Diagnosis | MCI | 0 | 0.1 | 489.31 | [-0.192, 0.193] |  |
| BNT | Risk × Diagnosis | AD | -0.886 | 0.2 | 444.74 | [-1.286, -0.486] |  |
| BNT | Risk × Diagnosis | NC - SCD | -0.071 | 0.08 | 364.33 |  | 0.783 |
| BNT | Risk × Diagnosis | NC - MCI | -0.065 | 0.12 | 467.34 |  | 0.943 |
| **BNT** | **Risk × Diagnosis** | **NC - AD** | **0.821** | **0.21** | **463.68** |  | **<.001** |
| BNT | Risk × Diagnosis | SCD - MCI | 0.006 | 0.11 | 474.21 |  | 1.000 |
| **BNT** | **Risk × Diagnosis** | **SCD - AD** | **0.892** | **0.21** | **456.07** |  | **<.001** |
| **BNT** | **Risk × Diagnosis** | **MCI - AD** | **0.886** | **0.23** | **446.18** |  | **<.001** |
| STT-A | Risk × Age | −1 SD | -0.029 | 0.07 | 464.59 | [-0.174, 0.115] |  |
| STT-A | Risk × Age | Mean | 0.122 | 0.06 | 474.2 | [-0.001, 0.245] |  |
| STT-A | Risk × Age | +1 SD | 0.273 | 0.07 | 475.53 | [0.134, 0.413] |  |
| Plasma Aβ42/40 ratio | Risk × Sex | Male | 0.167 | 0.11 | 353.87 | [-0.049, 0.383] |  |
| Plasma Aβ42/40 ratio | Risk × Sex | Female | -0.019 | 0.09 | 358.94 | [-0.186, 0.147] |  |
| **Plasma Aβ42/40 ratio** | **Risk × Sex** | **Male - Female** | **0.187** | **0.09** | **253.58** |  | **0.043** |

NOTE: Follow-up analyses were conducted for statistically significant Risk × moderator interactions identified in the Type III tests (Table S2). For continuous moderators, simple-slope estimates at −1 SD, the mean, and +1 SD are shown in bold. For categorical moderators, simple slopes are reported for each group, and statistically significant Tukey-adjusted pairwise comparisons are shown in bold. All continuous variables, including risk scores, age, education, and outcome measures, were standardized; sex and baseline diagnosis were sum-coded. p values for pairwise comparisons were adjusted using Tukey correction.
